# Identifying Candidate Protein Markers of Acute Kidney Injury in Acute Decompensated Heart Failure

**DOI:** 10.3390/ijms23021009

**Published:** 2022-01-17

**Authors:** Evelyn M. Templeton, Moritz Lassé, Torsten Kleffmann, Leigh J. Ellmers, Suetonia C. Palmer, Trent Davidson, Nicola J. A. Scott, John W. Pickering, Christopher J. Charles, Zoltan H. Endre, Vicky A. Cameron, A. Mark Richards, Miriam T. Rademaker, Anna P. Pilbrow

**Affiliations:** 1 Christchurch Heart Institute, Department of Medicine, University of Otago, Christchurch 8014, New Zealand; m.lasse@uke.de (M.L.); leigh.ellmers@otago.ac.nz (L.J.E.); nicola.scott@otago.ac.nz (N.J.A.S.); john.pickering@otago.ac.nz (J.W.P.); chris.charles@otago.ac.nz (C.J.C.); vicky.cameron@otago.ac.nz (V.A.C.); mark.richards@nus.edu.sg (A.M.R.); miriam.rademaker@otago.ac.nz (M.T.R.); anna.pilbrow@otago.ac.nz (A.P.P.); 2 Research Infrastructure Centre, Division of Health Sciences, University of Otago, Dunedin 9016, New Zealand; torsten.kleffmann@otago.ac.nz; 3 Department of Medicine, University of Otago, Christchurch 8014, New Zealand; suetonia.palmer@otago.ac.nz; 4 Department of Anatomical Pathology, Prince of Wales Hospital, Sydney, NSW 2031, Australia; trent.davidson@unsw.edu.au; 5 Department of Nephrology, Prince of Wales Hospital, Sydney, NSW 2031, Australia; zoltan.endre@unsw.edu.au; 6 Cardiovascular Research Institute, Department of Cardiology, National University of Singapore, Singapore 119077, Singapore

**Keywords:** acute kidney injury, acute decompensated heart failure, biomarker, proteomics, heart failure, SWATH–MS, acute renal failure, CCT6A, TRiC, CCT

## Abstract

One-quarter of patients with acute decompensated heart failure (ADHF) experience acute kidney injury (AKI)—an abrupt reduction or loss of kidney function associated with increased long-term mortality. There is a critical need to identify early and real-time markers of AKI in ADHF; however, to date, no protein biomarkers have exhibited sufficient diagnostic or prognostic performance for widespread clinical uptake. We aimed to identify novel protein biomarkers of AKI associated with ADHF by quantifying changes in protein abundance in the kidneys that occur during ADHF development and recovery in an ovine model. Relative quantitative protein profiling was performed using sequential window acquisition of all theoretical fragment ion spectra–mass spectrometry (SWATH–MS) in kidney cortices from control sheep (*n* = 5), sheep with established rapid-pacing-induced ADHF (*n* = 8), and sheep after ~4 weeks recovery from ADHF (*n* = 7). Of the 790 proteins quantified, we identified 17 candidate kidney injury markers in ADHF, 1 potential kidney marker of ADHF recovery, and 2 potential markers of long-term renal impairment (differential abundance between groups of 1.2–2.6-fold, adjusted *p* < 0.05). Among these 20 candidate protein markers of kidney injury were 6 candidates supported by existing evidence and 14 novel candidates not previously implicated in AKI. Proteins of differential abundance were enriched in pro-inflammatory signalling pathways: glycoprotein VI (activated during ADHF development; adjusted *p* < 0.01) and acute phase response (repressed during recovery from ADHF; adjusted *p* < 0.01). New biomarkers for the early detection of AKI in ADHF may help us to evaluate effective treatment strategies to prevent mortality and improve outcomes for patients.

## 1. Introduction

Heart failure (HF) affects more than 60 million people worldwide [1], and is associated with high mortality and morbidity [2]. Approximately one-quarter of patients with acute decompensated HF (ADHF) experience acute kidney injury (AKI) [3,4]—a complex disorder defined as acute kidney damage and/or an abrupt loss of kidney function. AKI complicating ADHF is associated with a near-doubling of death in patients with HF after one year [5,6], and efforts to improve the treatment of AKI in ADHF have been unsuccessful to date [6]. Prophylactic measures—including care in drug selection and dosing, and control of blood pressure—are valuable, but there is no proven specific pharmacotherapy for AKI [7]. This is in large part due to the failure to detect AKI at a stage where injury might be remediated. Early detection of AKI is clearly vital for timely introduction of effective interventions, and will remain crucial once effective agents are identified.

In the search for diagnostic and prognostic markers of AKI, numerous markers of renal cellular damage have been identified [8,9,10]; these include neutrophil gelatinase-associated lipocalin (NGAL), interleukin-18 (IL-18), liver-type fatty acid-binding protein (L-FABP), tissue inhibitor of metalloproteinases type 2 (TIMP-2), insulin-like growth factor-binding protein 7 (IGFBP7), kidney injury molecule-1 (KIM-1) and proenkephalin (PENK). Urinary TIMP-2 and IGFBP7, in combination (NephroCheck, Astute Medical Inc., USA), have FDA approval for evaluating the risk of moderate-to-severe AKI [11]. Other markers that have entered clinical practice include L-FABP, used in Japan to detect AKI in patients with liver failure [9], and NGAL, which is available for use in some European jurisdictions, as dictated by local policies [12]. Relatively few studies have explored these markers in ADHF [13,14,15,16,17], and the AKINESIS trial—the largest of such studies to date—showed no advantage of NGAL over plasma creatinine as a marker of AKI in ADHF [18].

While the utility of TIMP-2, IGFPB7, and L-FABP for the prediction of AKI within 1–7 days of ADHF onset has been investigated [13,14,15,16,17], no markers have sufficient sensitivity and specificity for routine clinical use, with several studies presenting mixed and inconclusive results [19]. This is in part due to variations in methodologies, heterogeneous study populations and clinical settings, and the lack of universal definitions of kidney impairment employed in research [19]. Consequently, the current body of evidence for the prognostic and diagnostic utility of various protein biomarkers for kidney injury in ADHF is insufficient to support their routine clinical use [19]. 

We aimed to identify novel candidate protein biomarkers of kidney injury, recovery, and long-term renal impairment in ADHF. We utilized an ovine model of pacing-induced ADHF, in which the development of ADHF induces an immediate decline in renal function that fails to fully resolve after approximately four weeks, and results in persistent structural/functional kidney injury [20] (for physiological characteristics in this model, see the Supplementary Materials, Table S1). Using mass spectrometry, we performed relative quantitative protein profiling to characterize the changes in protein abundance in the kidneys that occur during ADHF development and recovery. This approach identified 20 candidate protein markers of kidney injury and repair in ADHF, including 6 putative candidates supported by existing evidence and 14 novel candidates not previously implicated in AKI.

## 2. Results

### 2.1. Physiological Results

**Figure 1:**
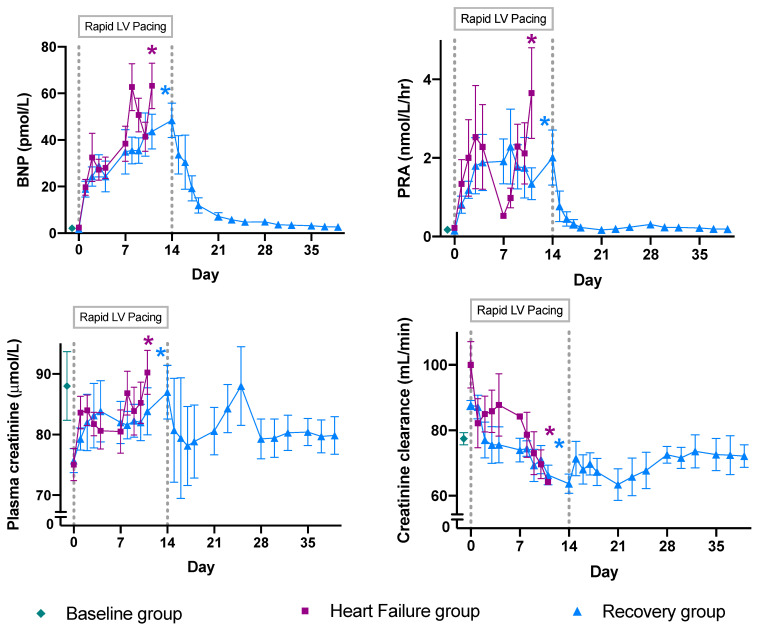
Mean ± standard error of the mean of serial physiological responses of sheep before and during the development of pacing-induced acute decompensated heart failure, and during 25 days of recovery following the cessation of pacing. Vertical dashed line at day = 0 represents the initiation of left ventricular pacing; vertical dashed line at day = 14 represents the termination of pacing. Sheep in the Heart Failure group were paced at 180 beats per minute (bpm) for 7 days, followed by pacing at 225 bpm for 4 days. Sheep in the Recovery group were paced at 220 bpm for 14 days. These data highlight the serial changes in physiological measures in response to the initiation and cessation of pacing within each group, with significant differences from day 0 shown by asterisks (paired-samples *t*-tests, *p* < 0.05). Pacing resulted in an increase in B-type natriuretic peptide concentration, an increase in plasma renin activity, an increase in plasma creatinine, and a decrease in creatinine clearance in both groups of paced animals (HF and Recovery). Although the HF and Recovery groups were exposed to slightly different pacing protocols, the degree of kidney injury was comparable between these groups, as shown by the lack of evidence for differences between the Heart Failure and Recovery groups using independent samples *t*-tests for mean BNP concentration, plasma creatinine concentration, and creatinine clearance at both day 0 and the final day of pacing (*p* > 0.14 for all tests). BNP: B-type natriuretic peptide; LV: left ventricular; PRA: plasma renin activity.

Physiological data for relevant endocrine and renal parameters are presented for the three groups—healthy control sheep (*n* = 5; “Baseline”), sheep with pacing-induced ADHF (*n* = 8; “Heart Failure”), and sheep after ~4 weeks recovery from ADHF (*n* = 7; “Recovery”)—in Figure 1. The pathophysiological effects of this model of ADHF have previously been well characterized [20], demonstrating a consistent association between induction of ADHF and the consequent development of renal dysfunction. Figure 1 displays the clinically relevant increase in B-type natriuretic peptide (BNP), plasma renin activity (PRA), and plasma creatinine concentrations, alongside a marked reduction in creatinine clearance over the pacing period within both paced groups (Heart Failure and Recovery; paired samples *t*-tests *p* < 0.05 for all comparisons). The degree of renal injury was comparable between the HF and Recovery groups, indicated by no differences in plasma creatinine concentration and creatinine clearance at either day 0 or the final day of pacing between the two paced groups (independent samples *t*-test *p* > 0.14 for all comparisons). However, there were striking differences between those sheep that underwent ~4 weeks of recovery compared to those euthanised at the HF timepoint. After recovery, we observed a return of the key HF biomarker BNP and a marker of HF-induced neurohormonal activation—PRA—to near-Baseline levels, indicating recovery from HF (day 0 versus last day of recovery; paired samples *t*-test *p* = 0.067 and *p* = 0.399, respectively). However, this model results in ongoing renal impairment that does not resolve upon recovery from HF. Creatinine clearance—a marker of renal function—does not return to Baseline levels upon cessation of pacing (day 0 versus final day of recovery; paired samples *t*-test *p* = 0.003).

### 2.2. Identifying Proteins of Differential Abundance

**Figure 2:**
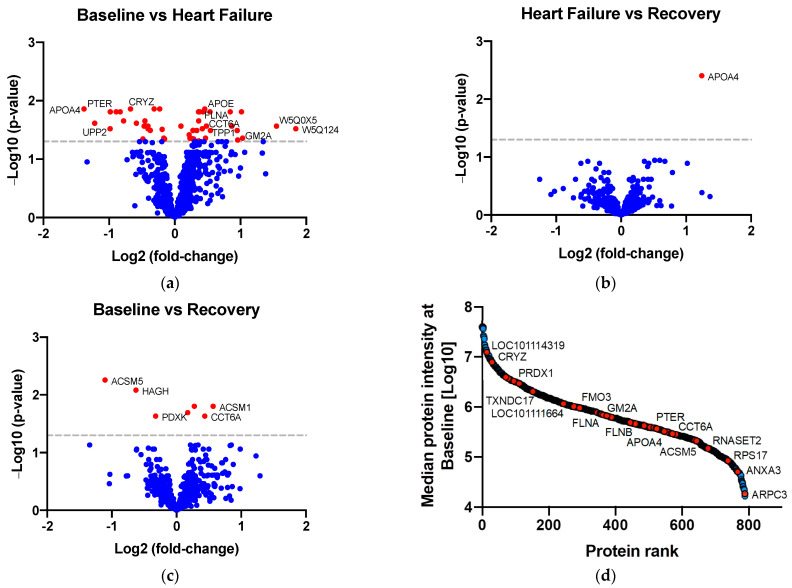
Volcano plots displaying −LOG_10_ of the Benjamini–Hochberg (BH) *p*-value against LOG_2_ of the protein fold-change for (**a**) Baseline vs. ADHF, (**b**) Heart Failure vs. Recovery, and (**c**) Baseline vs. Recovery. Red data points in the volcano plots indicate a BH *p*-value < 0.05; blue data points indicate a BH *p*-value ≥ 0.05. The horizontal dashed line represents *p* = 0.05. (**d**) In total, 790 proteins were quantified across four orders of magnitude of mass spectrometry signal. Proteins of differential abundance between timepoints with a fold-change ≥1.2 and Benjamini–Hochberg adjusted *p*-value < 0.05 are coloured red; proteins with a fold-change < 1.2 and/or an adjusted *p*-value ≥ 0.05 are coloured blue.

Of the 790 proteins robustly quantified in sheep kidney cortices (Figure 2), 52 were of differential abundance between timepoints (defined as a *p*-value adjusted for multiple comparisons <0.05; Figure 2). Proteins of differential abundance were categorized into three groups based on their pattern of abundance across the ADHF time course: (1) proteins that differed from Baseline to ADHF, but not from ADHF to Recovery (potential markers of kidney injury in ADHF); (2) proteins that differed from Baseline to ADHF and from ADHF to Recovery (kidney proteins associated with ADHF recovery); and (3) proteins that differed in abundance between Baseline and Recovery (potential markers of long-term renal impairment). A total of 48 proteins differed between Baseline and ADHF, 1 protein differed between ADHF and Recovery, and 7 proteins differed between Baseline and Recovery. Of these 52 proteins, 45 had a fold-change of ≥1.2 (Table 1; a ≥1.2-fold threshold was selected, as it is not practical to further investigate all proteins). For a full list of all protein fold-changes across timepoints and abundance at Baseline, see Supplementary Data.

Of the 45 proteins that differed by ≥1.2-fold between the timepoints (*p*-values < 0.05), 41 proteins were altered between Baseline and ADHF, 1 protein was altered between ADHF and Recovery, and 6 proteins were altered between Baseline and Recovery (Supplementary Materials, Figure S1). There was a small amount of overlap of proteins that changed by at least 1.2-fold between each of the timepoints, indicating that the abundance of a proportion of proteins may continue to be altered across ADHF development and recovery.

### 2.3. Pathway Activation and Inhibition

**Figure 3:**
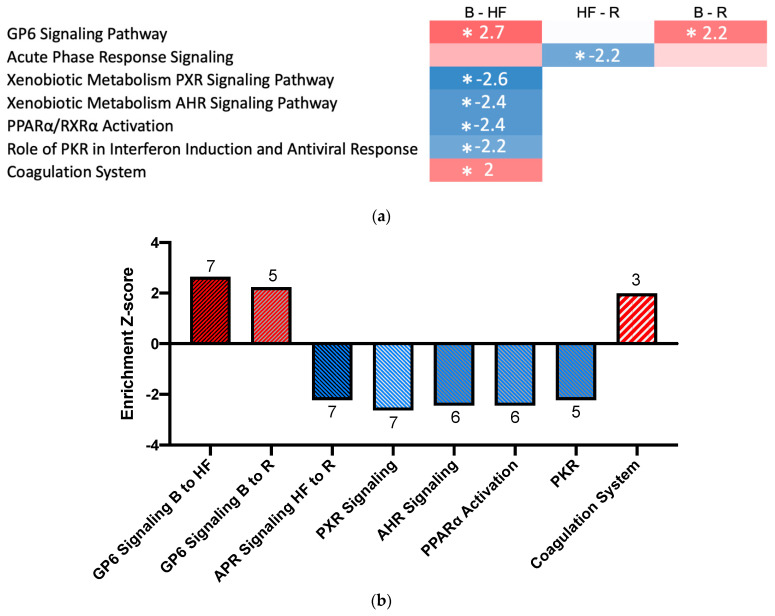

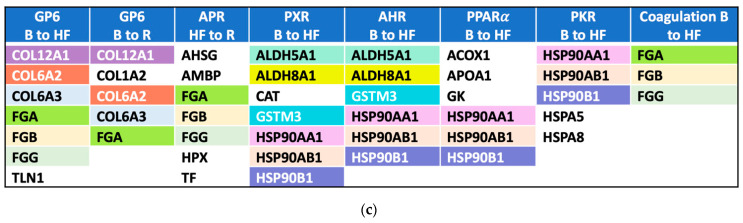
(**a**) Heatmap of pathway enrichment and activation/inhibition. Enrichment Z-scores are displayed in each box; red indicates a positive Z-score (activation of a pathway) while blue represents a negative Z-score (repression of a pathway). The intensity of colour represents the magnitude of the Z-score, with darker hues indicating greater scores. Starred timepoints indicate an absolute Z-score > 2 or < −2 and an adjusted *p*-value < 0.01, representing enriched and activated/repressed pathways. (**b**) Enrichment Z-scores for activated/repressed pathways. The number of proteins that caused annotation to the pathway is noted directly above/below each bar. Red signifies activation of a pathway, whilst blue indicates repression. (**c**) Lists of proteins that caused annotation to the enriched and activated/repressed pathways. Colours indicate when a protein has been annotated to more than one pathway. AHR: aryl hydrocarbon receptor signalling pathway, APR: acute phase response signalling pathway, B: Baseline, GP6: glycoprotein 6 signalling pathway, HF: Heart Failure, PKR: protein kinase R pathway, PPARα: peroxisome proliferator-activated receptor alpha, PXR: pregnane X receptor signalling pathway, R: Recovery.

Comparative pathway analysis was performed to investigate the molecular pathways altered in the kidneys in response to ADHF development and recovery. Of the 790 proteins quantified, 665 mapped to genes in the Ingenuity Pathway Analysis (IPA) database, and could be included in the analysis. Proteins altered across the timepoints were enriched for seven molecular pathways (absolute Z-score > 2 or < −2 and *p* < 0.01 after adjustment for multiple comparisons; Figure 3; for full pathways refer to the Supplementary Materials, Figure S2). These included two pathways with established roles in AKI [28,29]: the glycoprotein VI (GP6) signalling pathway (pro-inflammatory, activated between Baseline and ADHF), and the acute phase response signalling pathway (pro-inflammatory, inhibited between ADHF and Recovery). Enriched pathways previously implicated in AKI that were repressed between Baseline and ADHF included the xenobiotic pregnane X receptor (PXR) signalling pathway (anti-inflammatory) [30,31], the xenobiotic metabolism aryl hydrocarbon receptor (AHR) signalling pathway (anti-proliferation, pro-inflammatory) [32,33], the peroxisome proliferator-activated receptor alpha (PPARα/RXRα) pathway (anti-inflammatory) [34,35], and the protein kinase R (PKR) in interferon induction and antiviral response pathway (pro-apoptotic, pro-inflammatory) [36,37]. The coagulation system, also implicated in AKI [38,39], was activated between Baseline and ADHF.

### 2.4. Biomarker Discovery

**Figure 4:**
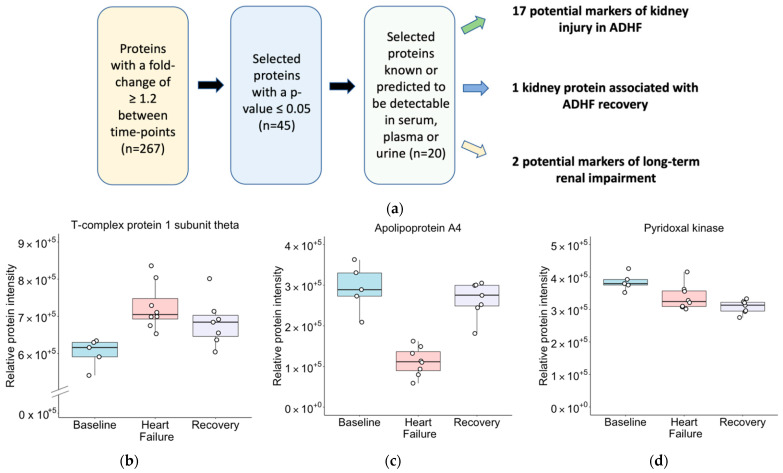
(**a**) Biomarker discovery workflow for candidate markers of renal impairment in ADHF, and (**b**–**d**) boxplots for proteins of differential abundance detectable in plasma, serum, or urine with adjusted *p*-values < 0.05 and fold-changes ≥1.2. Representative examples of (**b**) proteins of differential abundance between Baseline and ADHF, but not between ADHF and Recovery F (potential markers of kidney injury in ADHF); (**c**) proteins of differential abundance between Baseline and ADHF, and between ADHF and Recovery (kidney proteins associated with ADHF recovery); (**d**) proteins of differential abundance between Baseline and Recovery (potential markers of long-term renal impairment).

To identify candidate biomarkers of AKI in ADHF, putative secreted proteins were identified from the entire dataset with the IPA software “Biomarker Filter” workflow and the Human Protein Atlas secretome [27,40] (proteinatlas.org) (Supplementary Data). Of the 45 proteins with a differential abundance of at least 1.2-fold (*p* < 0.05), 20 were predicted to be detectable in serum, plasma, or urine, and were short-listed as potential circulating and/or excreted markers of AKI in ADHF (Table 2; Figure 4a). These proteins were subsequently categorized into three groups based on their pattern of abundance across the ADHF time course. This resulted in the identification of 17 potential markers of kidney injury in ADHF, 1 kidney protein associated with ADHF recovery, and 2 potential markers of long-term renal impairment (Figure 4b–d; Supplementary Materials, Figure S3). To look for previous associations with AKI, these candidate markers were searched across two large databases containing processed datasets: the IPA knowledgebase (https://www.qiagenbioinformatics.com/products/ingenuity-pathway-analysis) (accessed on 16 September 2021), and Harmonizome [26] (see Supplementary Methods for details). The shortlisted candidate biomarkers included 6 proteins supported by existing evidence, along with 14 novel candidates not previously associated with AKI (Table 2).

To assess the associations between these 20 candidate protein markers and known risk markers for AKI, correlation matrices were plotted (Supplementary Materials, Figure S4). At Baseline, heat shock protein 90 beta family 1 and T-complex protein 1 subunit gamma (CCT3) were positively correlated with plasma renin activity, peroxiredoxin was positively correlated with creatinine clearance, and CCT3 was positively correlated with plasma creatinine. During ADHF, GM2 ganglioside activator and crystallin zeta (CRYZ) were positively associated with plasma creatinine, and pyridoxal kinase was positively correlated with creatinine clearance. CRYZ remained positively associated with plasma creatinine upon recovery from ADHF.

## 3. Discussion

This study aimed to identify candidate protein biomarkers of kidney injury during ADHF, recovery from ADHF, and long-term renal impairment after ADHF, using an established ovine model of pacing-induced ADHF. SWATH–MS identified 20 proteins that differed in abundance in the kidneys in response to the development of and/or recovery from ADHF, and that may be detectable in biofluids, 14 of which had not been associated with AKI previously. These included 17 putative markers of kidney injury (differential abundance between Baseline and ADHF), 1 putative kidney marker of recovery from ADHF (differential abundance between Baseline and ADHF, and between ADHF and Recovery), and 2 putative markers of long-term renal impairment (differential abundance from Baseline after 4 weeks of Recovery). SWATH–MS is a hypothesis-free method of protein quantification that is not limited by prior knowledge. Therefore, these data represent the outcome of a novel, proteome-wide analysis of moderate–high-abundance proteins altered in the kidneys during the development of and recovery from ADHF, for the prioritization of candidate diagnostic and prognostic markers in patients with AKI and ADHF.

The three top candidate proteins across the proposed marker types (based on magnitude of fold-change between timepoints) were GM2 ganglioside activator protein (GM2AP encoded by the *GM2A* gene; putative marker of kidney injury in ADHF), apolipoprotein A4 (APOA4 encoded by *APOA4*; putative kidney marker of recovery from ADHF), and chaperonin-containing TCP1 subunit 6A (CCT6A encoded by *CCT6A*; putative long-term renal impairment marker). APOA4 is a lipid-binding protein synthesized in the small intestine, and is involved in numerous physiological processes, including lipid absorption and metabolism, platelet aggregation and thrombosis, anti-atherosclerosis, and glucose homeostasis [41]. High plasma concentrations of APOA4 have been correlated with mild-to-moderate renal failure, and may be a predictor of the progression of kidney disease [24,25]. GM2AP is a small glycoprotein normally found in the lysosome that acts as an important cofactor for the enzyme β-hexosaminidase in its degradation of GM2 ganglioside [42]. GM2AP has been associated with tubular damage [43], and its urinary concentrations have been shown to increase in AKI due to defective tubular reabsorption of the filtered protein [23]. In combination with chaperonin-containing TCP1 subunit 7, concentrations of GM2AP can predict whether patients with AKI will effectively recover their pre-AKI renal function [23].

Unlike APOA4 and GM2AP, the protein CCT6A has not previously been associated with AKI. CCT6A is a component of a molecular chaperone complex known as cytosolic chaperonin-containing t-complex polypeptide 1 (CCT, also known as the chaperonin-containing T-complex; TRiC), which guides the folding of proteins such as actin and tubulin [44]. CCT consists of eight paralogous subunits encoded by *TCP1*, *CCT2*, *CCT3*, *CCT4, CCT5*, *CCT6A*, *CCT6B*, *CCT7*, and *CCT8*, which together form two back-to-back rings [45]. All subunits are necessary for the functioning of CCT, but each makes unique contributions to a range of cellular functions [46]. For example, CCT6 plays an essential role in the folding of actin and tubulin, and has been shown to have pathophysiological involvement in fibrous disease [46], whereas CCT3 influences cellular proliferation and apoptosis [47]. In the present study, CCT6A increased 1.4-fold (*p* < 0.05) between Baseline and ADHF, and remained elevated after 4 weeks of Recovery (1.4-fold relative to Baseline, *p* < 0.05) suggesting that it may be a marker of long-term renal impairment. Notably, three other proteins from the CCT chaperone complex family were also among the top candidate biomarkers in this study: T-complex protein 1 subunit gamma (CCT3), T-complex protein 1 subunit theta (CCT8), and T-complex protein 1 subunit alpha (TCP1). Therefore, the molecular chaperone complex CCT may play an important role in the pathogenesis of AKI following ADHF. This hypothesis is supported by previous studies that implicate urinary concentrations of the CCT subunit CCT7 (also known as TCP1-*eta*) as a marker of renal tubular cell damage [48] and patient prognosis in AKI, in combination with GM2AP [23]. In the present study, CCT7 displayed a 1.25-fold increase between Baseline and ADHF (adjusted *p*-value = 0.06).

The observed changes in renal protein abundances reflect pathways relevant to the inflammatory component of AKI pathogenesis. AKI is characterized by endothelial injury and activation, resulting in inflammation, tubular epithelial cell death, and haemostasis [28,49]. The GP6 signalling pathway, which our data suggest was activated during the development of ADHF, is a pro-inflammatory pathway involved in the early stages of AKI [28]. The injury and subsequent activation of renal endothelial cells result in the binding of GP6 to platelet-activating endothelial cell components, initiating platelet activation and adhesion [28]. In addition, our data show that this pathway may also be persistently activated in the kidneys during recovery from AKI (enriched and activated between Baseline and Recovery). In addition, the acute phase response (APR) signalling pathway—a pro-inflammatory pathway involved in the immune response to tissue damage (such as that occurring in AKI) [29]—was repressed during recovery from ADHF. Through the production of pro-inflammatory mediators with high cytotoxic potential (such as chemokines, cytokines, free radical species, and enzymes), the APR pathway can amplify and extend cell death and damage, consolidating and worsening organ dysfunction following injury [29]. Components of the APR that decreased in abundance between Baseline and HF in the current study include hemopexin, alpha-2-HS-glycoprotein, fibrinogen alpha chain, fibrinogen beta chain, fibrinogen gamma chain, and transcription factor A (mitochondrial). Fibrinogen is a clotting factor that is upregulated in the kidneys in AKI [50], whilst hemopexin is an acute phase reactant that binds and neutralizes free haem [51]. Because of this binding, hemopexin can decrease haem-driven oxidative stress and mitigate tissue injury during haemolytic states [51]. These changes in protein abundances may reflect the switch from inflammation towards healing and repair in the kidney, as evidenced by the activation and repression of different pro-inflammatory pathways at different stages throughout the development of and recovery from ADHF. 

Protein abundance data were compared to transcriptomic data previously acquired using the same ovine model of ADHF [20], and 5 of the top 20 protein markers were corroborated by these gene expression data. The genes encoding CCT6A, filamin A, filamin B, heat shock protein 90 β family member 1 (HSP90B1),and pyridoxal kinase were altered in kidney tissue during the development of and/or recovery from ADHF by ≥1.2-fold (adjusted *p*-value < 0.01). Furthermore, the direction and magnitudes of fold-changes of the gene transcripts were largely concordant with those observed for the protein products (Supplementary Materials, Table S2 and Figure S5)—for example, in the present study, a 1.4-fold increase in the *CCT6A* gene transcript was observed between Baseline and ADHF, and a 1.4-fold increase in CCT6A protein was observed between Baseline and ADHF.

Thus far, research into candidate protein biomarkers in the specific context of AKI following ADHF has produced mixed and inconclusive results, in part due to the complex and potentially confounding factors inherent in observational patient cohorts [19]. A strength of the present study is the elimination of confounding factors such as age, gender, ethnicity, comorbidities, and decongestive therapy through the use of an established ovine model of ADHF [52,53]. This animal model simplifies the identification of proteins altered in the kidneys in response to kidney injury following ADHF, although it is not without limitations. Animal models do not reflect the entire complexity of ADHF observed in human patients, especially in terms of the chronic nature of the underlying HF, which undergoes acute decompensation, the antecedent substrates of HF (including hypertension and arterial disease), and other comorbidities that frequently affect elderly patients [54]. Our animal model did not receive standard ADHF medications that would have better reflected the clinical setting [54]. Finally, proteins in this study were quantified from kidney cortical tissue, rather than plasma. Whilst putative secreted proteins were shortlisted as candidate biomarkers, direct empirical evidence is still required in order to confirm whether the changes in abundance observed in the kidneys are also reflected and quantifiable in the circulation and or urine.

This study has a number of limitations. First, the renal cortex contains a mixture of cell types, including blood vessels, glomeruli, and convoluted tubules, meaning that the observed changes in protein abundance could be derived from any of these cell types. Nevertheless, regardless of cellular origin, proteins derived from the renal cortex that differ in concentration between those with and without kidney injury represent potential candidate markers. Microdissection could be applied in future to characterize the origins of candidate proteins and identify glomerular or tubule-specific markers. Second, a sham group was not able to be included in the study due to ethical reasons, and it is possible that a degree of renal impairment resulted from the anaesthetic and surgical procedures used on the animals. To control for this, animals were allowed to recover for at least 14 days following surgery, so as to allow time for the potential effects on renal function to attenuate before experimental measures were initiated. Third, the degree of renal injury was not identical across the two paced groups of sheep, due to slightly differing pacing protocols. Despite this, the injury was comparable, with both groups showing consistent changes across the pacing period that represented physiologically significant renal impairment. Finally, the candidate markers identified will ultimately require validation in human samples in order to demonstrate their clinical utility. Our findings provide early evidence to support further investigation of these novel candidate protein markers in the clinical context of AKI following ADHF.

In summary, this study identified several candidate protein markers of kidney injury in ADHF, including both candidates supported by existing evidence and additional candidates not previously implicated in AKI. Reliable detection of early kidney injury in ADHF could enable more timely implementation of supportive measures (such as avoidance of hypotension and nephrotoxic drugs) and inform trial design for the testing of new renoprotective agents to ultimately improve patient outcomes [55]. Further investigation of circulating and excreted levels of these candidate proteins in patients with ADHF is needed in order to evaluate their diagnostic and prognostic utility.

## 4. Materials and Methods

### 4.1. Animals

Experiments were approved by the Animal Ethics Committee of the University of Otago Christchurch (No. C23/10 and AUP-19-77; in accordance with the New Zealand Animal Welfare Act 1999 and Amendment No. 2 (2015)), and conformed to the current National Institute of Health guidelines (8th edition).

#### 4.1.1. Study Protocol for Control Sheep

Adult Coopworth ewes (*n* = 5; “Baseline” group; 2–5 years; Lincoln University Farm, Canterbury, New Zealand) were housed in a metabolic crate for 24 h to allow for urine collection. During this time, sheep had free access to food and water. After 24 h, urine was collected (for measurement of volume and creatinine concentrations) and blood samples were taken from the jugular vein (for measurement of BNP, PRA, creatinine concentrations) before the sheep were euthanised via intravenous anaesthetic overdose (150 mg/kg pentobarbital). Kidney cortex samples were excised immediately post-mortem, snap-frozen in liquid nitrogen, and stored at −80 °C for subsequent proteomic analysis (Supplementary Materials, Figure S6).

#### 4.1.2. Instrumentation of Paced Sheep

Adult Coopworth ewes (*n* = 15; 4–6 years; 41–69 kg; Lincoln University Farm, Canterbury, New Zealand) were anaesthetized and instrumented via a left lateral thoracotomy, as previously described in [20,56]. Briefly, a 7-French His-bundle electrode was stitched sub-epicardially to the left ventricle (LV) to enable subsequent cardiac pacing, and other instrumentation was performed to allow for blood and haemodynamic sampling. Following instrumentation, animals recovered for at least 14 days before beginning the study protocol. Throughout the investigation, sheep were held in metabolic crates with free access to water, and received a diet of food pellets and Lucerne chaff (providing ~75 mmol sodium/day; 150 mmol potassium/day).

#### 4.1.3. Study Protocol for Paced Sheep

In *n* = 8 sheep (“Heart Failure” group), rapid LV pacing at 180 beats per minute (bpm) was carried out for 7 days to produce a state of mild compensated HF (days 0–7), followed by pacing at 225 bpm for 4 days to induce ADHF (days 7–11), as previously described in [56] (Figure S6). In *n* = 7 sheep (“Recovery” group), animals underwent 14 days of rapid LV pacing at 220 bpm to induce ADHF, followed by termination of pacing and 25 days of recovery [20] (Supplementary Materials, Figure S6). Serial haemodynamic, blood, and urine sampling were performed throughout each protocol. [20]. Creatinine clearance (mL/min) was calculated as urine creatinine concentration × volume/plasma creatinine.

This model of ADHF is characterized by a decrease in cardiac output, widespread neurohormonal activation, sodium and water retention, development of pulmonary oedema and congestion, decline in arterial pressure, and increases in cardiac pre-load and systemic peripheral resistance [20,52]. Importantly, the development of ADHF in this model is consistently associated with renal dysfunction—paced sheep display increased plasma creatinine concentrations and reduced creatinine clearance during ADHF; following 25 days of recovery, creatinine clearance remains reduced [20].

#### 4.1.4. Specimen Preparation, Handling, and Storage

Blood samples for assay of plasma hormones (ANP, BNP, PRA) and biochemical measurements (creatinine) were drawn into tubes on ice, centrifuged at 4 °C for 5 min, and then stored at −80 °C until analysed. Urine was collected and stored at −20 °C until analysed. Kidney cortex samples were rapidly collected at the end of each study protocol, immediately following euthanasia (150 mg/kg IV pentobarbital 300) and snap-frozen in liquid nitrogen before storage at −80 °C for subsequent mass spectrometry analysis. These cortex samples contained a mixture of cell types, including glomeruli, convoluted tubules, and blood vessels.

### 4.2. Sample Preparation for Proteomics

The Sample Preparation by Easy Extraction and Digestion (SPEED) protocol was performed as described by Doellinger et al. [57]. Briefly, 100 mg of ground, frozen kidney cortex was dissolved in trifluoroacetic acid (TFA; Sigma-Aldrich, St. Louis, MO, USA) at a 1:4 *v*/*v* sample:TFA ratio. Reduction was performed using Tris(-carboxyethyl)phosphine (TCEP; Sigma-Aldrich, St. Louis, MO, USA) at a final concentration of 10 mM, and alkylation was simultaneously performed using 2-chloroacetamide (CAA; Sigma-Aldrich, St. Louis, MO, USA) at a final concentration of 40 mM. Protein concentration was determined using turbidity as described by Doellinger et al., and an aliquot of 150 µg of protein was adjusted to 0.75 µg/µL. Digestion was performed using a protein:trypsin ratio of 25:1 (sequencing-grade modified trypsin, PRV5111, Promega Fitchburg, WI, USA) at 37 °C for 20 h. Peptides were desalted using reverse-phase Vydac C18 Silica 96-well MACROspin plates (SNS SS18V-L, The Nest Group Inc., Ipswich, MA, USA), as detailed in the Supplementary Methods.

### 4.3. Mass Spectrometry

Retention time calibration peptides (iRT-Kit, Biognosys, Schlieren, Switzerland) were spiked into each sample at 75× dilution of the iRT stock solution. SWATH–MS was carried out on a 5600+ triple-TOF mass spectrometer coupled to an Ekspert NanoLC 415 system (Eksigent, AB Sciex, Dublin, CA, USA) equipped with an in-house packed emitter tip column filled with 2.6 µm Aeris C18 material (Phenomenex, Torrance, CA, USA) on a length of 20 cm. Peptides were separated over a 120-minute gradient using a binary solvent system (solvent A: 1% ACN, 0.1% FA in water, solvent B: 90% ACN, 0.1% FA in water). To generate spectral libraries, the mass spectrometer was run in data-dependent acquisition (DDA) mode (technical details in Supplementary Methods). SWATH–MS spectra were acquired over a 2-hour gradient using variable window width for precursor ion selection (technical details in Supplementary Methods). Each sample was injected three times, and the median ion intensity was calculated across the three SWATH–MS runs.

### 4.4. SWATH Data Processing

DDA data files were used to generate a spectral reference library in ProteinPilot (v4.5, AB Sciex), as described in Supplementary Methods. At a false discovery rate of 1.0%, this library contained 1777 proteins. SWATH–MS data were analysed using the SWATH Acquisition™ MicroApp in PeakView software (v2.2, AB Sciex). To correct for possible retention time shifts between SWATH–MS runs and library spectra, the ion signals of Biognosys peptides and endogenous high-abundance proteins consistently detected across samples were aligned (peptide sequences provided in Supplementary Methods). The SWATH Acquisition™ MicroApp processing settings were set as follows: 6 peptides per protein, 6 transitions per peptide, a 12-minute window for the extracted ion chromatogram, FDR threshold of 1.0%, a mass accuracy of 75 ppm, and a fixed library rank of peptides. Extracted transition ion, peptide, and protein peak areas were exported in Excel format for quality control processing and statistical analysis.

### 4.5. Quality Control

Ion intensity data were processed in R [58,59]. Normalization was carried out by multiplying each sample by the ratio of the mean ion intensity of all samples (area under the curve, AUC) to the AUC of the individual sample. Ions were excluded from a sample if their intensity was below the lower detection limit (calculated for each ion as Q_1_—(1.5 × IQR). Ions were excluded across the entire dataset if the coefficient of variation (CV) for technical injection replicates was >25 in at least 20% of samples. The median intensity of each ion was selected and summed to the corresponding peptide area, and then peptide areas were summed to protein areas [60,61,62].

### 4.6. Statistical Analysis

All statistical analyses were performed using R [58,59]. Three separate Welch one-way ANOVA tests were performed using the *tableone* R package (version 0.13.0) [63] to identify proteins of differential abundance between timepoints (Baseline vs. ADHF; ADHF vs. Recovery; Baseline vs. Recovery). *p*-Values were adjusted using the Benjamini–Hochberg method to decrease the false discovery rate, and fold-changes were reported.

### 4.7. Comparison with AKI-Related Genes

Because it was not practical to further investigate all proteins, we chose proteins that differed between Baseline and ADHF, ADHF and Recovery, and Baseline and Recovery (fold-change ≥1.2, adjusted *p*-value < 0.05). These proteins were compared with 884 genes associated with AKI, as recorded in two large databases: the Ingenuity Pathway Analysis (IPA; Qiagen Inc., https://www.qiagenbioinformatics.com/products/ingenuity-pathway-analysis) (accessed on 16 September 2021) knowledgebase (*n* = 94), and Harmonizome [26] (*n* = 814; 24 genes included in both databases; see Supplementary Methods).

### 4.8. Identifying Enriched and Activated/Repressed Pathways

The core and comparative gene set enrichment analysis workflow in Ingenuity Pathway Analysis software (Qiagen Inc., accessed on 16 September 2021) was used to test for enrichment of canonical pathways. Z-scores were used to determine the net effect of individual gene expression changes on a pathway—that is, whether the combined effect of independent gene expression alterations predicted activation or repression of a pathway. Z-scores > 2 indicate pathway activation, while Z-scores <−2 indicate pathway repression. A *p*-value of *p* < 0.01 after correction for multiple comparisons was used for all IPA analyses.

### 4.9. Identifying Circulating or Urinary Biomarkers

Potential circulating or urinary biomarkers were identified using (1) the IPA software’s “Biomarker Filter” workflow, which annotates proteins that have been experimentally observed in the biofluids of choice based on current literature (primarily [64,65]), and (2) the Human Protein Atlas secretome (proteinatlas.org), which includes 2641 proteins with a signal peptide and no transmembrane-spanning region, annotated with plasma concentrations where available (bioinformatics approach). These potential circulating or urinary biomarkers were compiled for comparison with proteins identified in the current study.

## Figures and Tables

**Table 1:** Proteins of differential abundance between timepoints with a fold-change ≥1.2 and Benjamini–Hochberg adjusted *p*-value < 0.05. B: Baseline, HF: Heart Failure, R: Recovery.

Protein Name	Gene	Timepoint	Fold-Change	Adjusted *p*-Value	Previously Associated with AKI	Enriched in Kidney Cells ^2^
Upregulated proteins						
Annexin	*ANXA3*	B-HF	↑ 1.37	0.01	Yes ^1^ [21]	
NTR domain-containing protein W5NUX8	*LOC101123672*	B-HF	↑ 1.79	0.02	No	
Uncharacterized protein W5P4A8	*RNASET2*	B-HF	↑ 2.02	0.02	No	
Filamin A	*FLNA*	B-HF	↑ 1.35	0.02	No	
Apolipoprotein E	*APOE*	B-HF	↑ 1.45	0.02	Yes ^1^ [22]	Enriched in proximal tubular cells
Talin 1	*TLN1*	B-HF	↑ 1.30	0.02	No	
T-complex protein 1 subunit delta	*CCT4*	B-HF	↑ 1.28	0.02	No	
Ribosomal protein S17	*RPS17*	B-HF	↑ 1.29	0.02	No	
Chaperonin containing TCP1 subunit 6A	*CCT6A*	B-HF	↑ 1.40	0.03	No	
		B-R	↑ 1.35	0.02		
SERPIN domain-containing protein W5Q0X5	LOC101115576	B-HF	↑ 2.92	0.03	No	
Actin-related protein 2/3 complex subunit 3	*ARPC3*	B-HF	↑ 1.83	0.03	No	
Filamin B	*FLNB*	B-HF	↑ 1.34	0.03	No	
SERPIN domain-containing protein W5Q124	LOC101119509	B-HF	↑ 3.58	0.03	No	
Integrin beta	*ITGB1*	B-HF	↑ 1.21	0.03	Yes ^1^	
Clusterin	*LOC101113728*	B-HF	↑ 1.93	0.03	No	
Tripeptidyl peptidase 1	*TPP1*	B-HF	↑ 1.46	0.03	No	
T-complex protein 1 subunit alpha	*TCP1*	B-HF	↑ 1.26	0.03	No	
Heat shock protein 90 beta family member 1	*HSP90B1*	B-HF	↑ 1.38	0.04	Yes^1^	
GM2 ganglioside activator	*GM2A*	B-HF	↑ 2.05	0.04	Yes ^1^ [23]	
T-complex protein 1 subunit theta	*CCT8*	B-HF	↑ 1.20	0.05	No	
Thioredoxin domain containing 17	*TXNDC17*	B-HF	↑ 1.22	0.05	No	
GC vitamin D binding protein	*GC*	B-HF	↑ 1.94	0.05	No	
T-complex protein 1 subunit gamma	*CCT3*	B-HF	↑ 1.29	0.05	No	
Profilin	*PFN1*	B-R	↑ 1.21	0.02	No	
Acyl-CoA synthetase medium chain family member 1	*ACSM1*	B-R	↑ 1.48	0.02	No	
Downregulated proteins						
Hydroxyacylglutathione hydrolase	*HAGH*	B-HF	↓ 1.60	0.01	No	Enriched in proximal tubular cells
		B-R	↓ 1.54	0.01		
Apolipoprotein A4	*APOA4*	B-HF	↓ 2.61	0.01	Yes ^1^ [24,25]	
		HF-R	↑ 2.36	0.004		
Crystallin zeta	*CRYZ*	B-HF	↓ 1.24	0.01	No	Enriched in distal tubular cells, proximal tubular cells, and collecting duct cells
Phosphotriesterase related	*PTER*	B-HF	↓ 1.97	0.02	No	Enriched in proximal tubular cells
Apolipoprotein A2	*APOA2*	B-HF	↓ 1.86	0.02	No	
Dimethylaniline monooxygenase [N-oxide-forming]	*FMO3*	B-HF	↓ 1.78	0.02	No	
Glutathione transferase	*LOC101111664*	B-HF	↓ 1.72	0.02	No	
Solute carrier family 27 member 2	*SLC27A2*	B-HF	↓ 1.37	0.02	Yes ^1^	Enriched in proximal tubular cells
Aldo_ket_red domain-containing protein	*LOC101109111*	B-HF	↓ 2.33	0.02	No	
Sulfurtransferase	*TST*	B-HF	↓ 1.50	0.02	No	Enriched in proximal tubular cells
Uncharacterized protein W5NYA7	*LOC101114319*	B-HF	↓ 1.38	0.03	No	
Solute carrier family 5 member 12	*SLC5A12*	B-HF	↓ 1.36	0.03	No	Enriched in proximal tubular cells
Acyl-CoA_dh_1 domain-containing protein W5Q8A9		B-HF	↓ 1.37	0.03	No	
Dehydrogenase/reductase 1	*DHRS1*	B-HF	↓ 1.33	0.03	No	
Uridine phosphorylase	*UPP2*	B-HF	↓ 1.98	0.03	No	Enriched in distal and proximal tubular cells
Uncharacterized protein W5PN31		B-HF	↓ 1.33	0.03	No	
Peroxiredoxin	*PRDX1*	B-HF	↓ 1.29	0.03	Yes ^1^	
3-hydroxybutyrate dehydrogenase 1	*BDH1*	B-HF	↓ 1.40	0.05	No	
Acyl-CoA synthetase medium chain family member 5	*ACSM5*	B-R	↓ 2.15	0.01	No	
Pyridoxal kinase	*PDXK*	B-R	↓ 1.25	0.02	No	

^1^ Previous associations determined by comparing proteins to a list of 884 genes associated with AKI sourced from two large databases: the IPA knowledgebase (https://www.qiagenbioinformatics.com/products/ingenuity-pathway-analysis) (accessed on 16 September 2021) and Harmonizome [26]; see Supplementary Methods for details. ^2^ Single-cell-type enrichment lists for proximal tubular cells, distal tubular cells, and collecting duct cells obtained from the Human Protein Atlas [27] (accessed on 3 November 2021). Arrows indicate the direction of the observed change in protein abundance.

**Table 2:** Proteins with a fold-change ≥ 1.2 and Benjamini–Hochberg adjusted *p*-values < 0.05 that are potentially detectable in the plasma, serum, or urine.

Protein Name	Gene	Timepoint	Fold-Change	Adjusted *p*-Value	Previously Associated with AKI
**Potential markers of kidney injury in ADHF**					
Upregulated					
Filamin A	*FLNA*	B-HF	↑ 1.35	0.02	No
Apolipoprotein E	*APOE*	B-HF	↑ 1.45	0.02	Yes ^1^ [22]
Talin 1	*TLN1*	B-HF	↑ 1.30	0.02	No
Filamin B	*FLNB*	B-HF	↑ 1.34	0.03	No
Integrin beta	*ITGB1*	B-HF	↑ 1.21	0.03	Yes^1^
Tripeptidyl peptidase 1	*TPP1*	B-HF	↑ 1.46	0.03	No
T-complex protein 1 subunit alpha	*TCP1*	B-HF	↑ 1.26	0.03	No
Heat shock protein 90 beta family member 1	*HSP90B1*	B-HF	↑ 1.38	0.04	Yes ^1^
GM2 ganglioside activator	*GM2A*	B-HF	↑ 2.05	0.04	Yes
T-complex protein 1 subunit theta	*CCT8*	B-HF	↑ 1.20	0.05	No
Thioredoxin domain containing 17	*TXNDC17*	B-HF	↑ 1.22	0.05	No
GC vitamin D binding protein	*GC*	B-HF	↑ 1.94	0.05	No
T-complex protein 1 subunit gamma	*CCT3*	B-HF	↑ 1.29	0.05	No
Downregulated					
Crystallin zeta	*CRYZ*	B-HF	↓ 1.24	0.01	No
Apolipoprotein A2	*APOA2*	B-HF	↓ 1.86	0.02	No
Uridine phosphorylase	*UPP2*	B-HF	↓ 1.98	0.03	No
Peroxiredoxin	*PRDX1*	B-HF	↓ 1.29	0.03	Yes^1^
**Kidney proteins associated with ADHF recovery**					
Apolipoprotein A4	*APOA4*	B-HF	↓ 2.61	0.01	Yes ^1^ [24,25]
		HF-R	↑ 2.36	0.004	
**Potential markers of long-term renal impairment**					
Chaperonin containing TCP1 subunit 6A	*CCT6A*	B-HF	↑ 1.40	0.03	No
		B-R	↑ 1.35	0.02	
Pyridoxal kinase	*PDXK*	B-R	↓ 1.25	0.02	No

^1^ Previous associations determined by comparing proteins to a list of 884 genes associated with AKI sourced from across two large databases: the IPA knowledgebase (https://www.qiagenbioinformatics.com/products/ingenuity-pathway-analysis) (accessed on 16 September 2021), and Harmonizome [26]; see Supplementary Methods for details. Arrows indicate the direction of the observed change in protein abundance.

## Data Availability

The mass spectrometry proteomics data have been deposited to the ProteomeXchange Consortium via the PRIDE partner repository, with the dataset identifier PXD028983.

## Supplementary Materials

The following are available online at https://www.mdpi.com/article/10.3390/ijms23021009/s1.
