# Supplementary material for: Identifying Candidate Protein Markers of Acute Kidney Injury in Acute Decompensated Heart Failure
Source: Int J Mol Sci. 2022 Jan 17;23(2):1009. doi: 10.3390/ijms23021009 (PMC8778509; doi:10.3390/ijms23021009)
Supplement: Supplementary file 1 [file ijms-23-01009-s001.zip › Supplementary Figures Templeton et al.pdf]

Supplementary Material

Table S1. Physiological characteristics of sheep in the "Recovery" group throughout the pacing and recovery protocol. Mean and standard deviations are reported at Baseline (Day 0), during pacing induced heart failure (Day 14) and during recovery (Day 39). Medians and interquartile ranges are presented for variables with skewed data. Paired sample t-test p-values are presented for the Pacing and Recovery timepoints relative to Baseline. For full physiological and histological reports see Rademaker et. al. 2021 [20].

	**Baseline (Day 0)**	**Pacing (Day 14)**	**p-value Baseline vs Pacing**	**Recovery (Day 39)**	**p-value Baseline vs Recovery**
Cardiac output (L/min)	7.52 (± 1.26)	3.75 (± 0.57)	<0.001	7.2 (± 0.96)	0.35
Mean arterial pressure (mmHg)	88.6 (± 6.39)	67.4 (± 5.52)	<0.001	87 (± 11.73)	0.40
Left atrial pressure (mmHg)	4.7 (± 1.41)	28.7 (± 3.9)	<0.001	5.9 (± 1.44)	0.018
Renal perfusion pressure (mmHg)	85.5 (± 6.03)	43.8 (± 4.2)	<0.001	83.6 (± 11.64)	0.34
B-type natriuretic peptide (pmol/L) †	2.3 (0.5 – 2.6)	47.9 (33 - 61.4)	<0.001	2.7 (2.0 – 3.8)	0.11
Atrial natriuretic peptide (pmol/L) †	17.4 (16.1 – 25.6)	399.6 (369.5 – 410.9)	<0.001	22.1 (20.8 – 28.6)	0.082
Creatinine clearance (mL/min)	91 (± 9)	65 (± 9)	0.001	74 (± 12)	0.003
Urine volume (L/24hrs)	1.2 (± 0.4)	0.68 (± 0.23)	0.001	1.8 (± 0.7)	0.062
Urine creatinine (mmol/hr)	0.41 (± 0.06)	0.34 (± 0.04)	0.002	0.36 (± 0.02)	0.004
Urine sodium (mmol/hr)	3.06 (± 1.5)	0.53 (± 0.14)	<0.001	2.7 (± 0.49)	0.203

† Median (IQR)

Figure S1. Venn diagram of proteins of differential abundance between timepoints with a fold-change ≥ 1.2 (adjusted $p < 0.05$). HF = Acute decompensated heart failure.

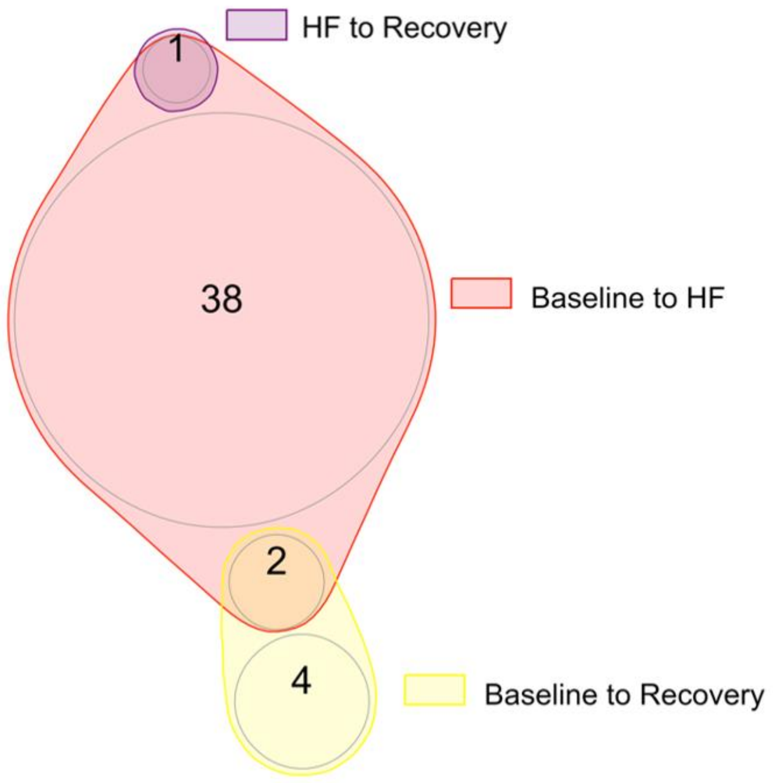

Figure S2. Enriched and activated/repressed gene signaling pathways

Figure S2A. Glycoprotein IV (GP6) gene signaling pathway illustrating altered abundance of individual proteins from Baseline to Heart Failure, Heart Failure to Recovery and Baseline to Recovery.

GP6
Baseline to Heart Failure

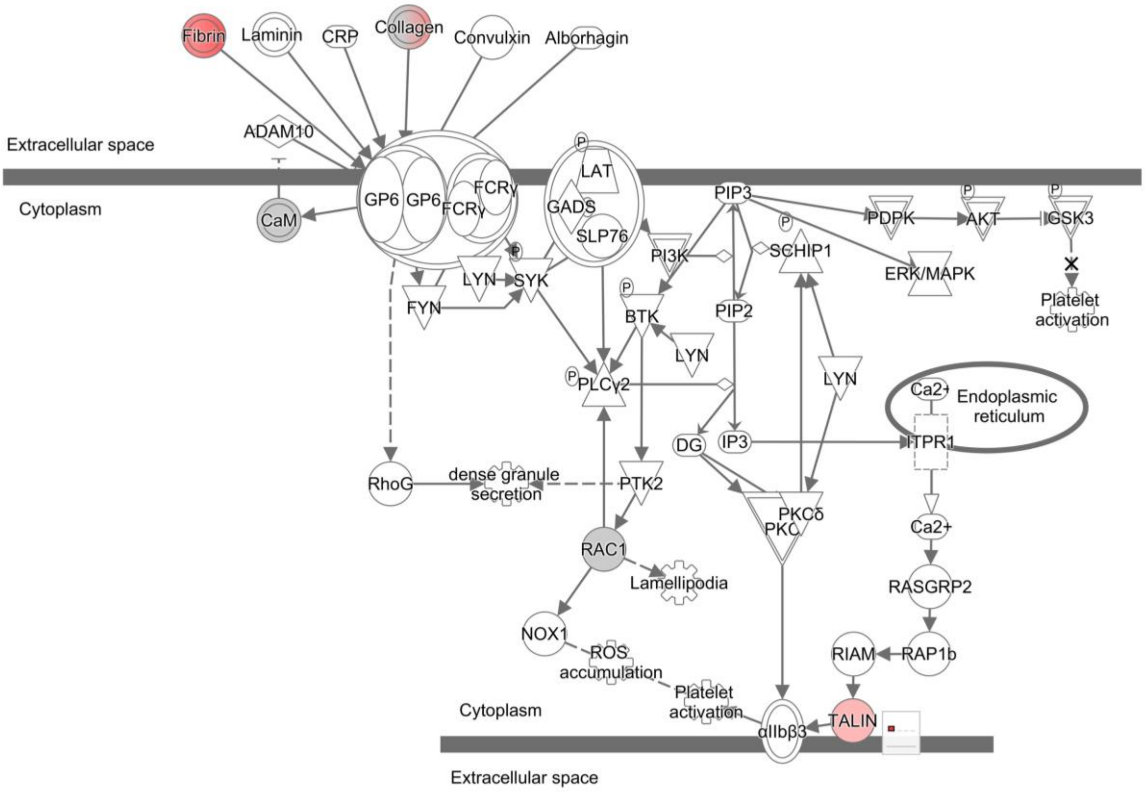

Heart Failure to Recovery

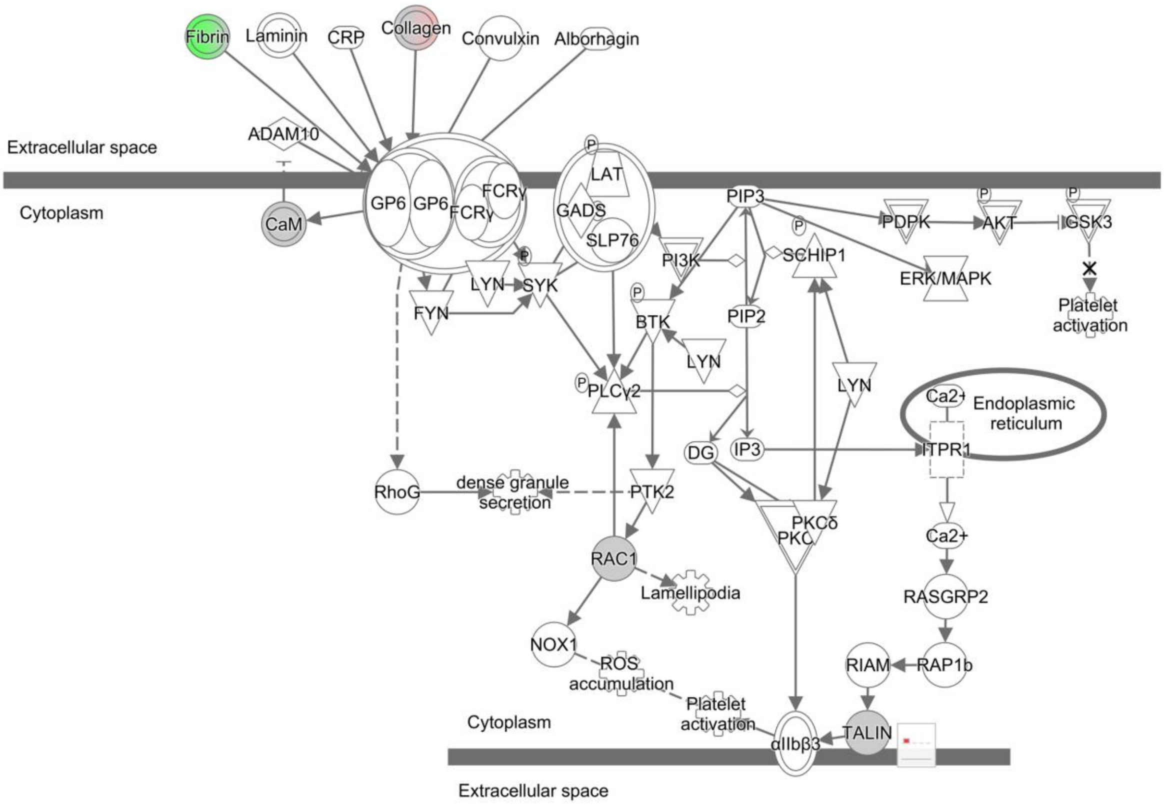

Red indicates increased abundance; green indicates decreased abundance

Baseline to Recovery

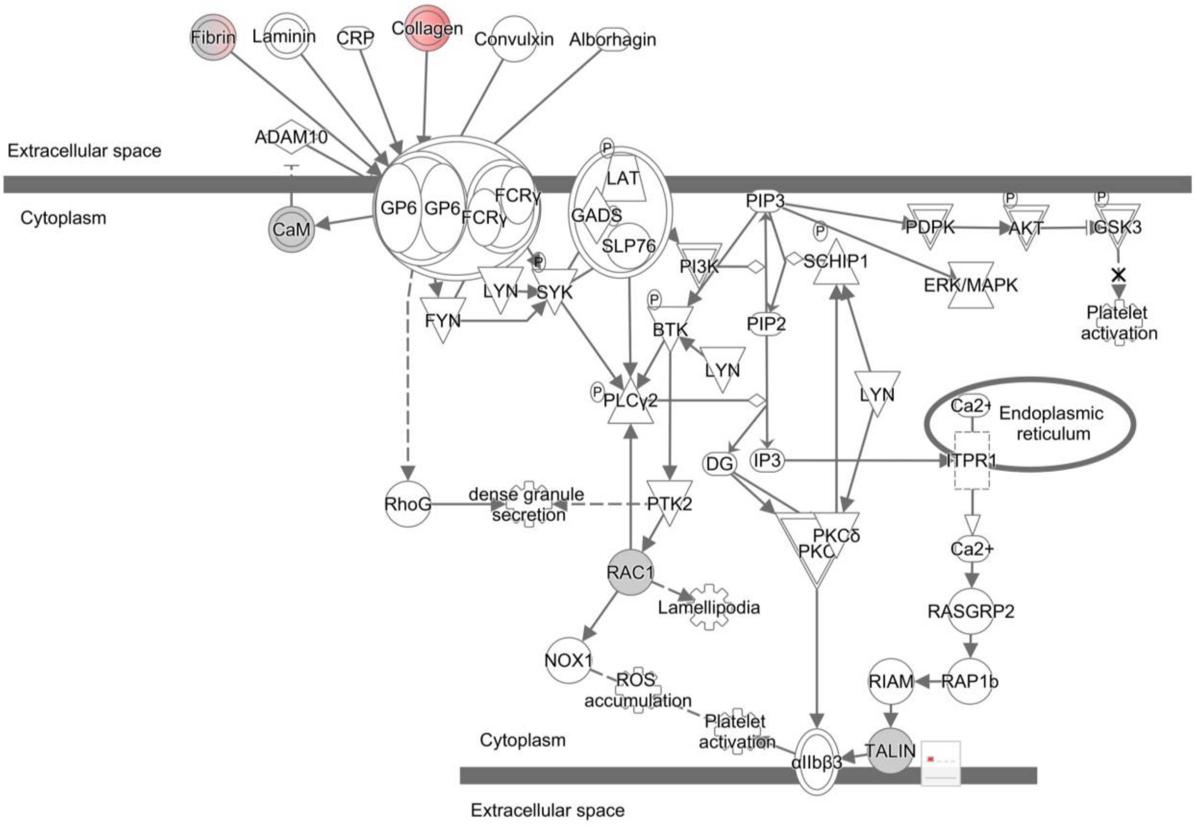

Red indicates increased abundance; green indicates decreased abundance

Figure S2B. Acute Phase Response (APR) gene signaling pathway illustrating altered abundance of individual proteins from Baseline to Heart Failure, Heart Failure to Recovery and Baseline to Recovery.

APR
Baseline to Heart Failure

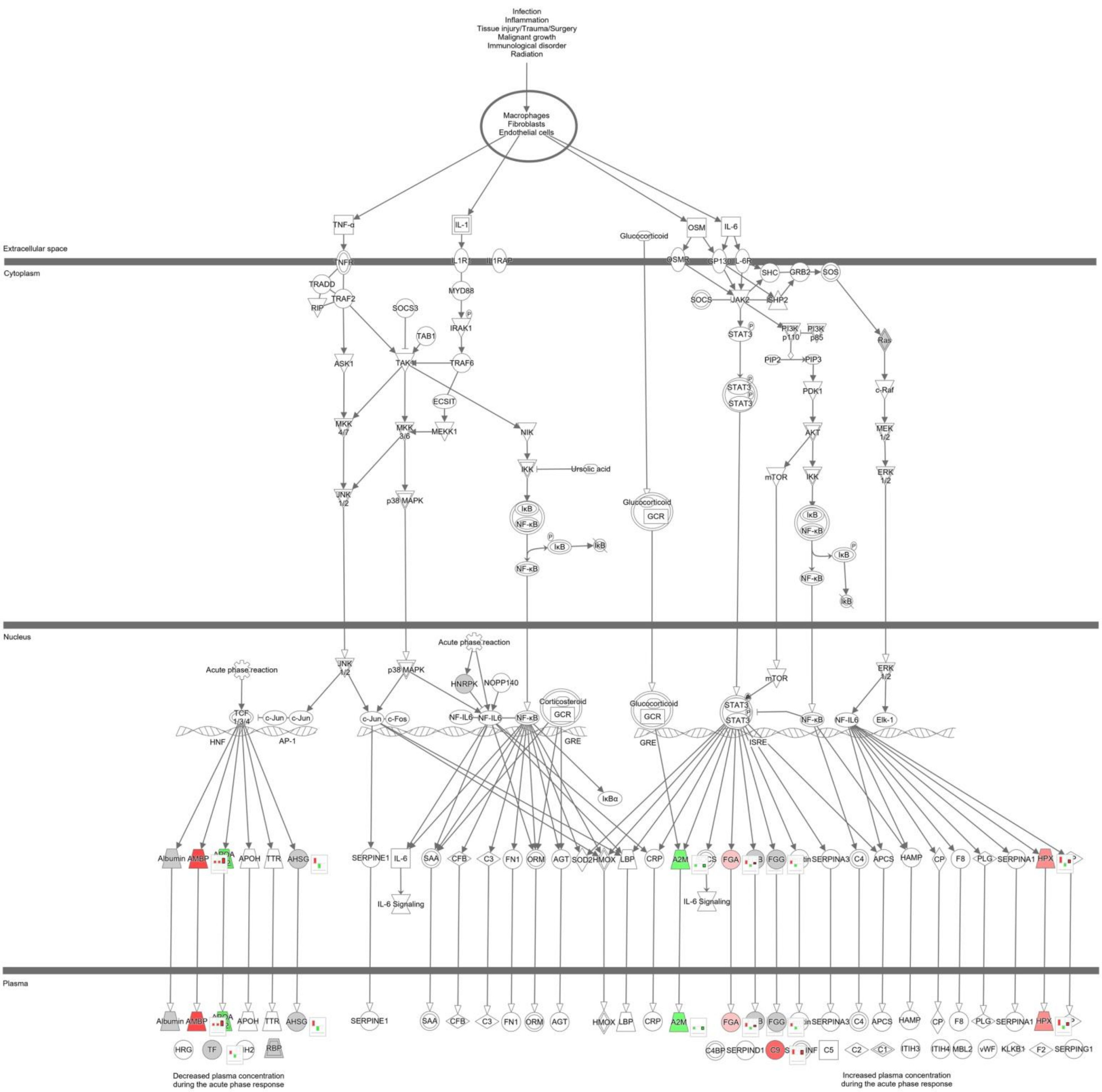

Red indicates increased abundance; green indicates decreased abundance

Heart Failure to Recovery

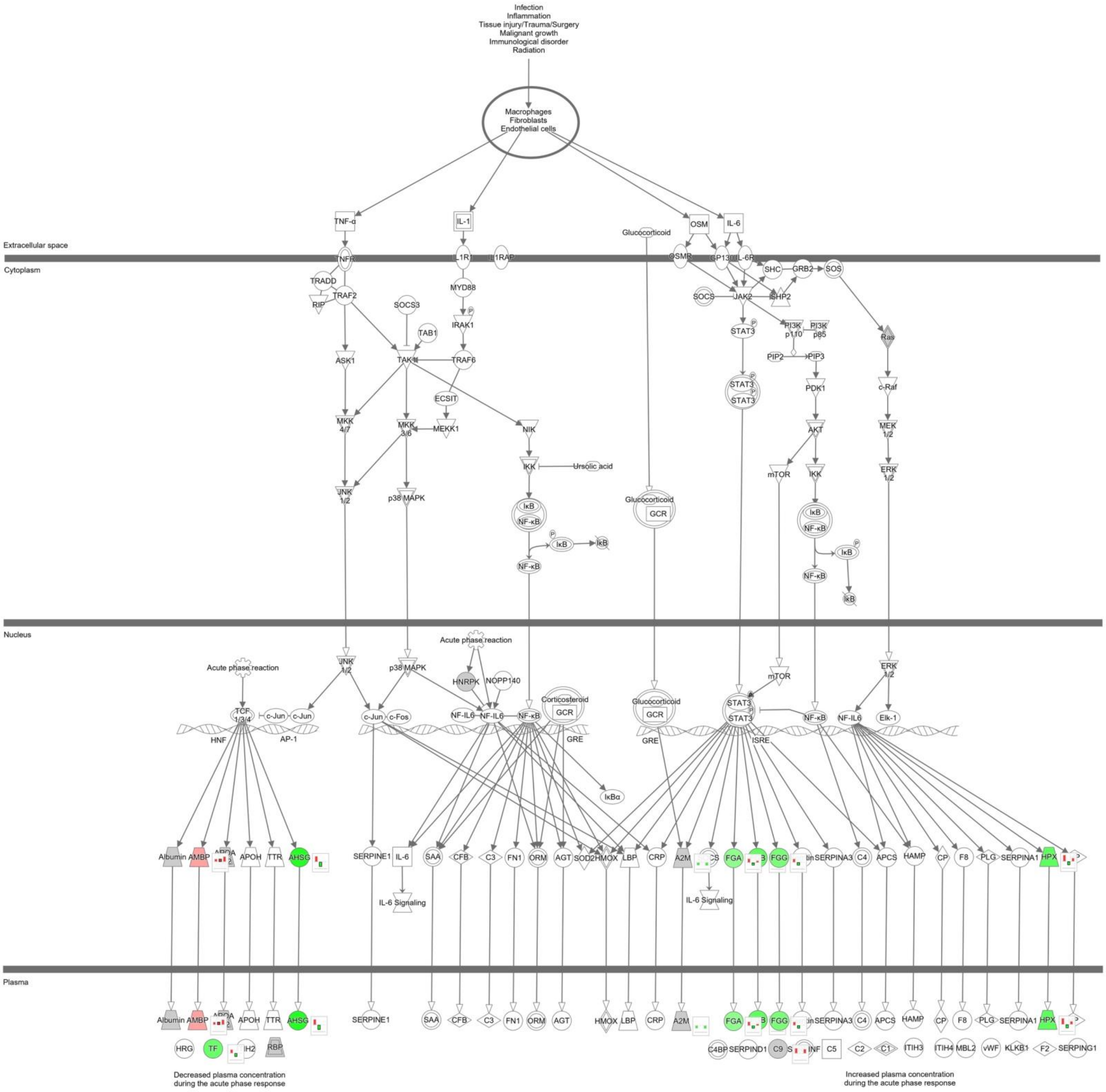

Red indicates increased abundance; green indicates decreased abundance

Baseline to Recovery

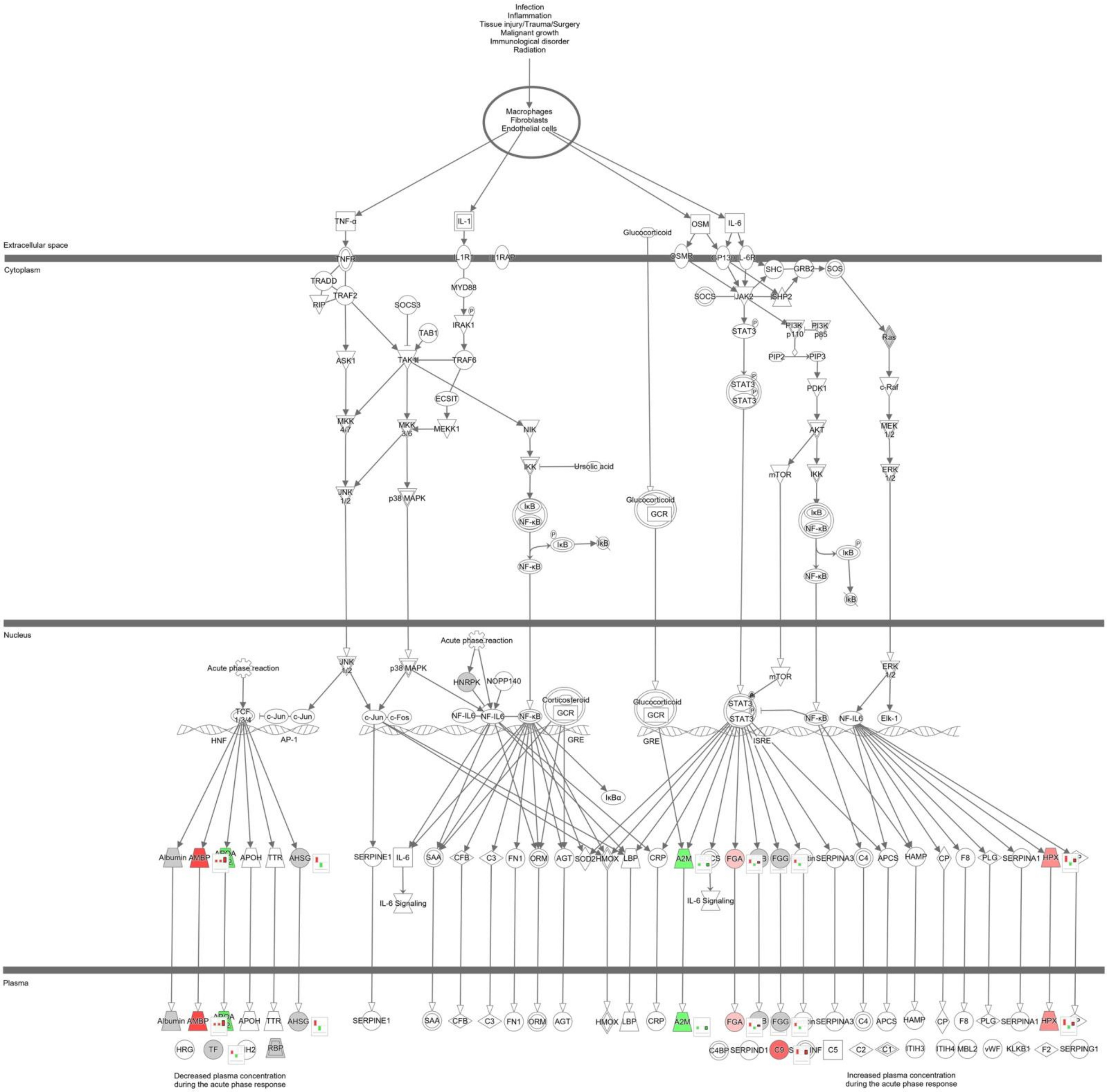

Red indicates increased abundance; green indicates decreased abundance

Figure S2C. Xenobiotic Metabolism Pregnane X Receptor (PXR) gene signaling pathway illustrating altered abundance of individual proteins from Baseline to Heart Failure.

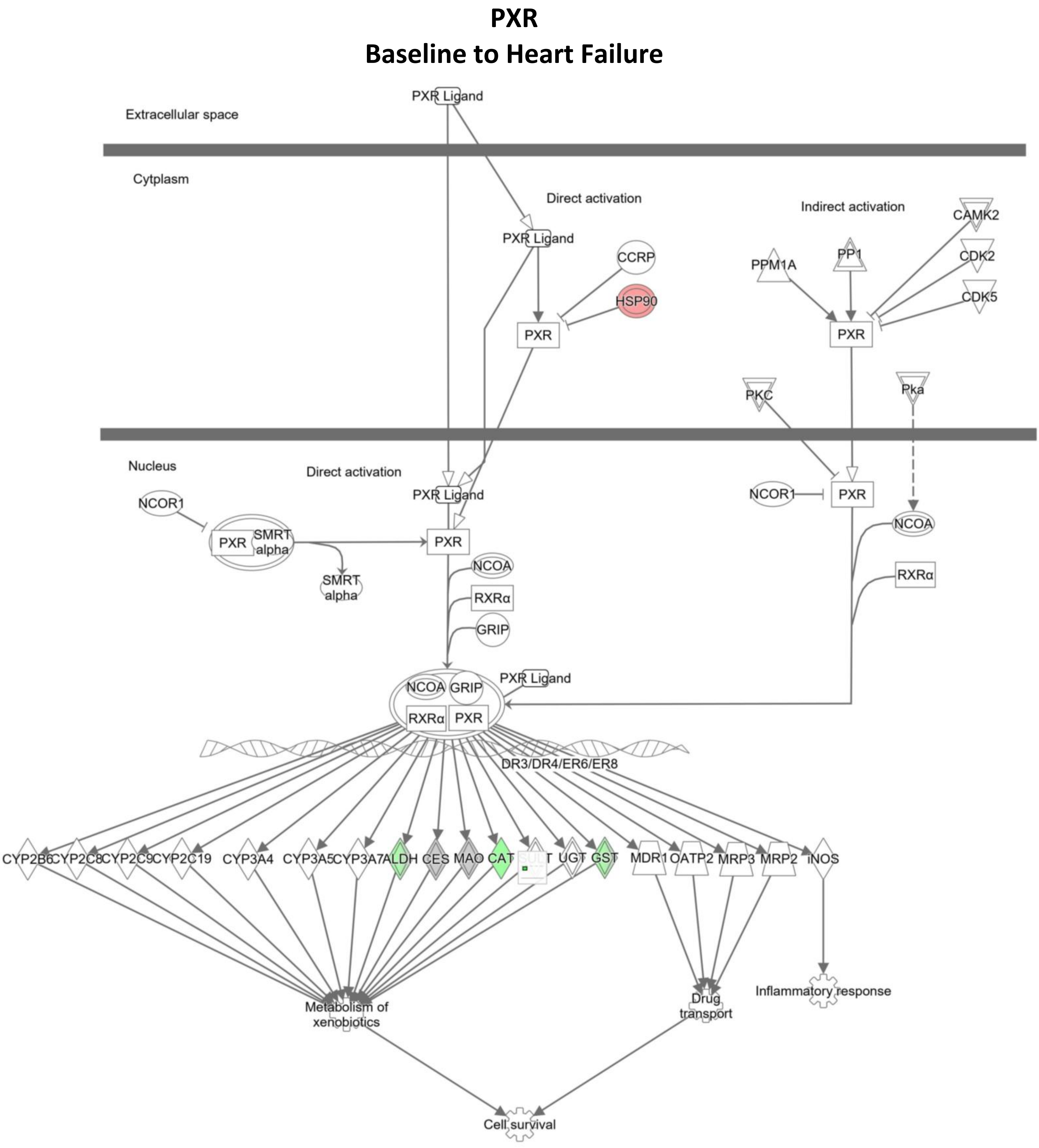

Red indicates increased abundance; green indicates decreased abundance

Figure S2D. Xenobiotic Metabolism Aryl hydrocarbon receptor (AHR) gene signaling pathway illustrating altered abundance of individual proteins from Baseline to Heart Failure.

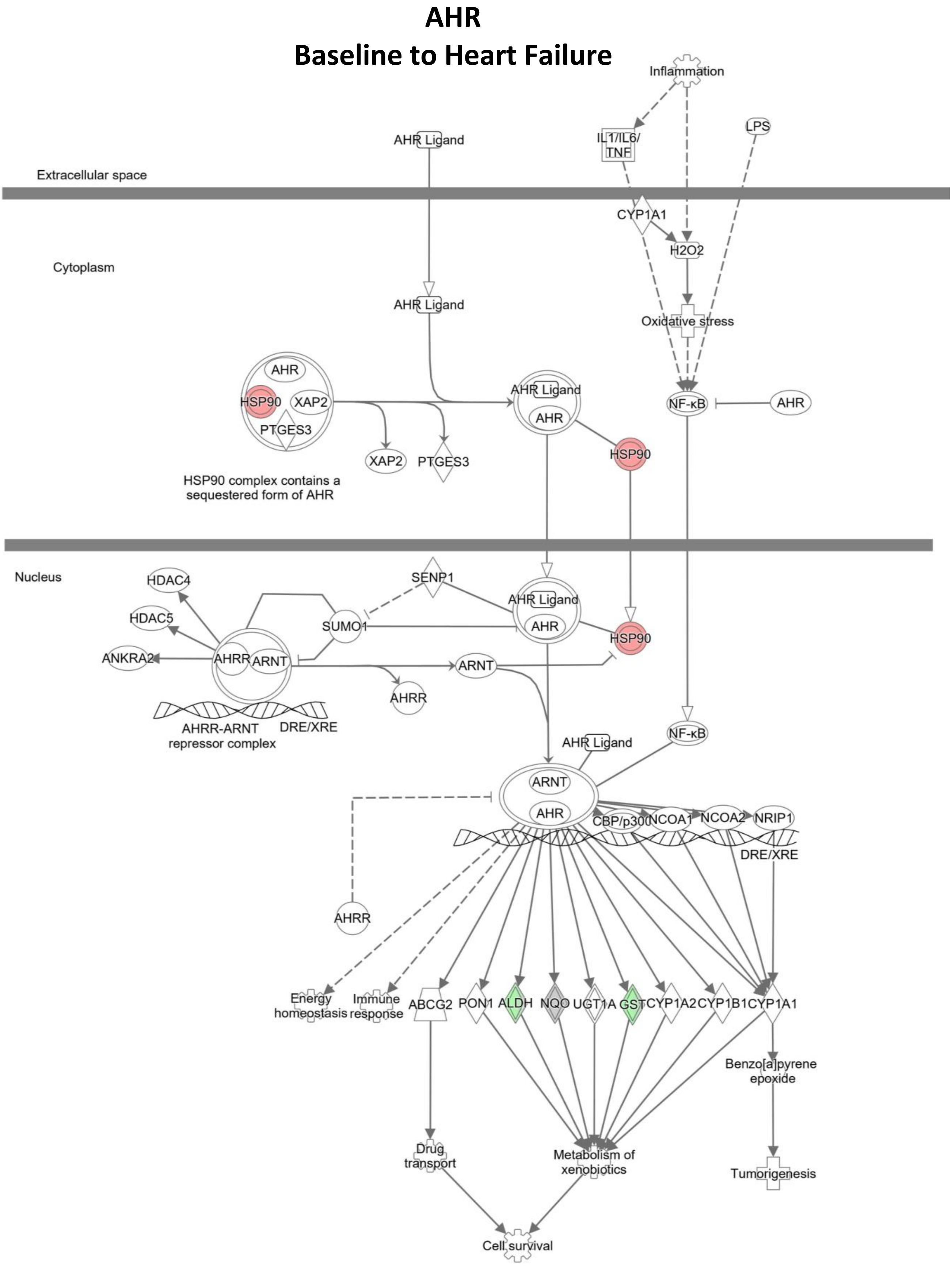

Red indicates increased abundance; green indicates decreased abundance

Figure S2E. Peroxisome Proliferator Activated Receptor alpha (PPARα/RXRα) gene signaling pathway illustrating altered abundance of individual proteins from Baseline to Heart Failure.

PPARα/RXRα
Baseline to Heart Failure

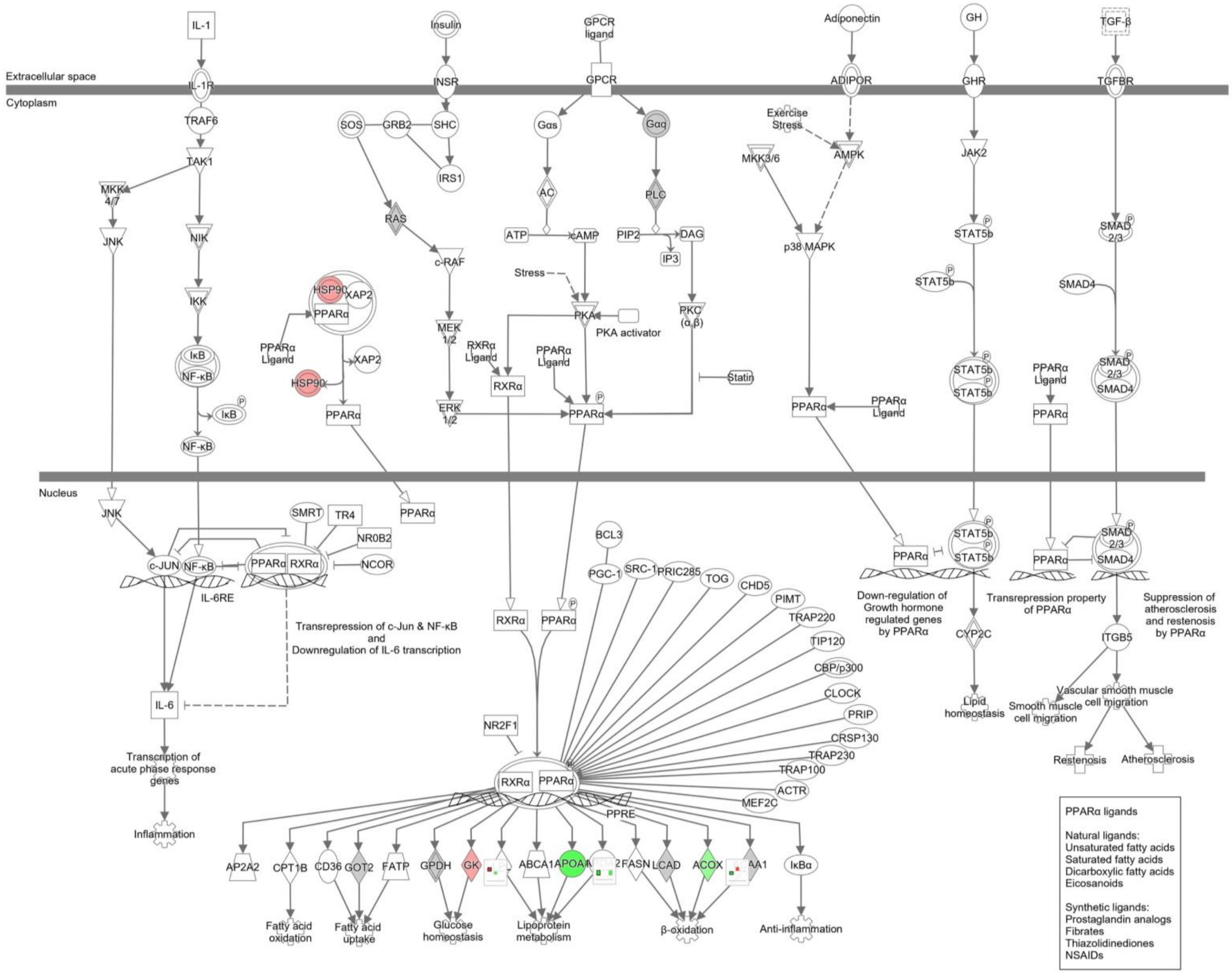

Red indicates increased abundance; green indicates decreased abundance

Figure S2F. Protein Kinase R (PKR) in the interferon and antiviral response gene pathway illustrating altered abundance of individual proteins from Baseline to Heart Failure.

PKR
Baseline to Heart Failure

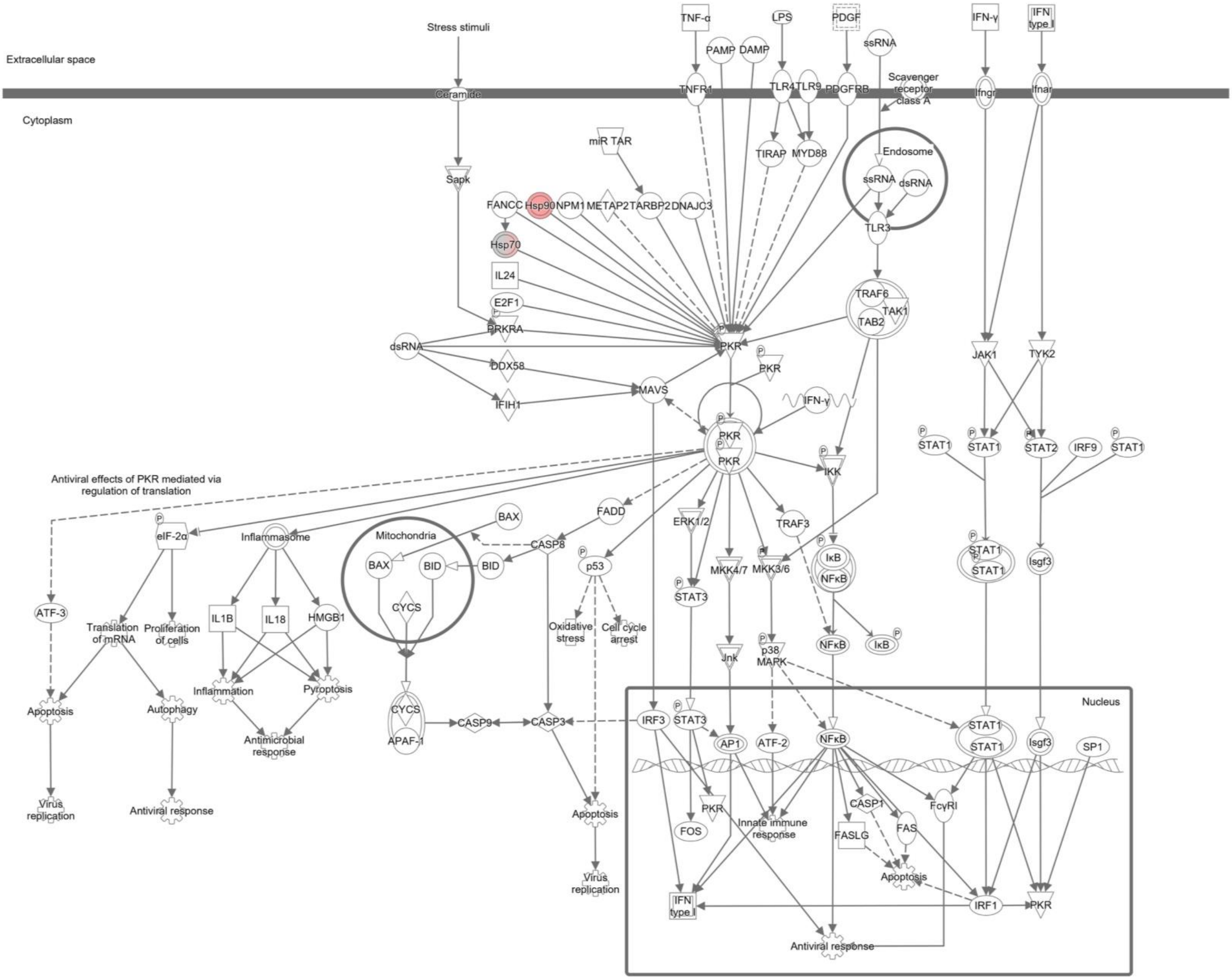

Red indicates increased abundance; green indicates decreased abundance

Figure S2G. Coagulation system gene pathway illustrating altered abundance of individual proteins from Baseline to Heart Failure.

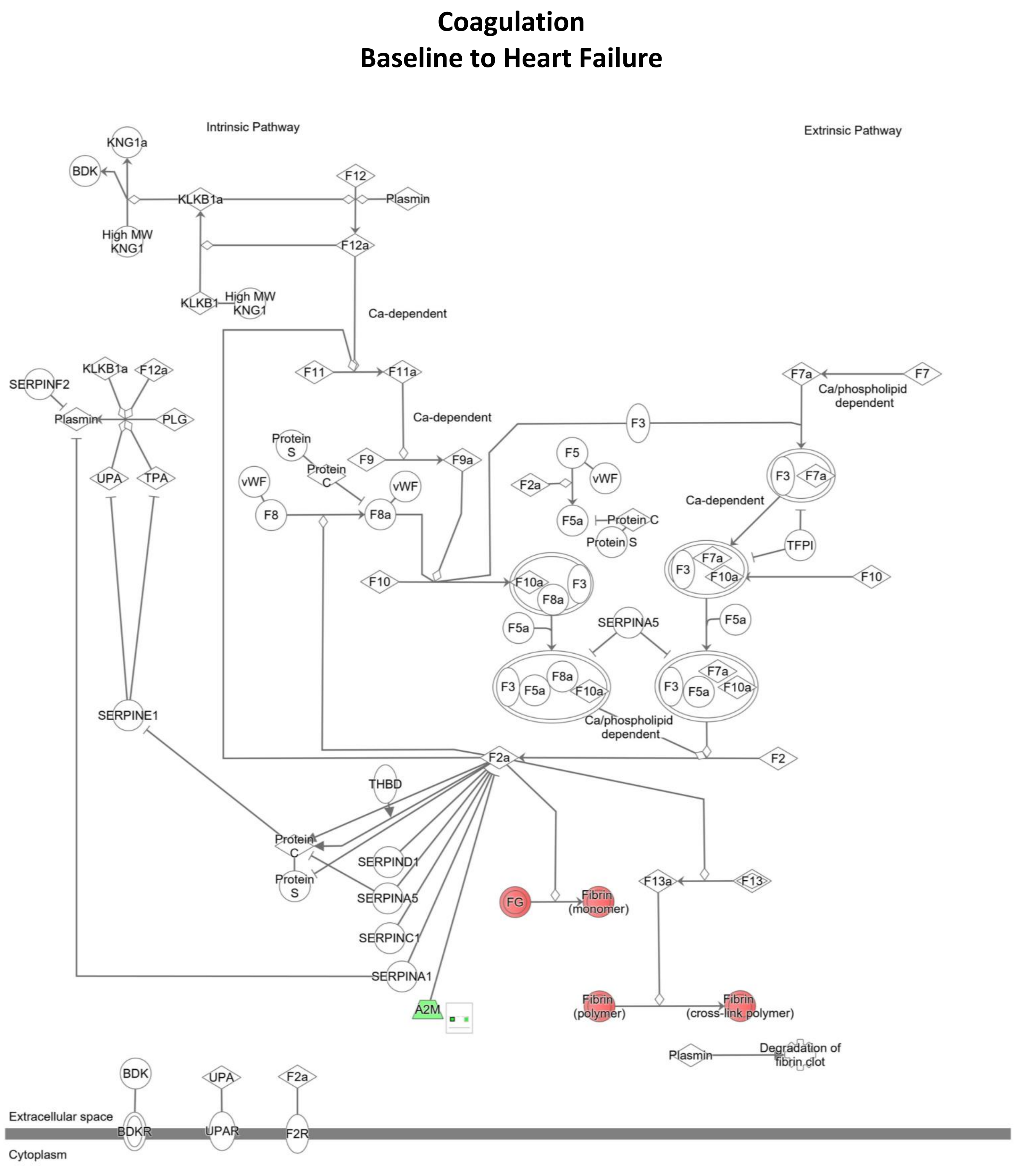

Red indicates increased abundance; green indicates decreased abundance

Figure S3. Box plots for proteins of differential abundance between any pair of timepoints (Baseline and ADHF, ADHF and Recovery form ADHF, Baseline and Recovery from ADHF) detectable in plasma, serum or urine with adjusted p-values < 0.05 and fold-changes ≥ 1.2. Whiskers represent the range of data.

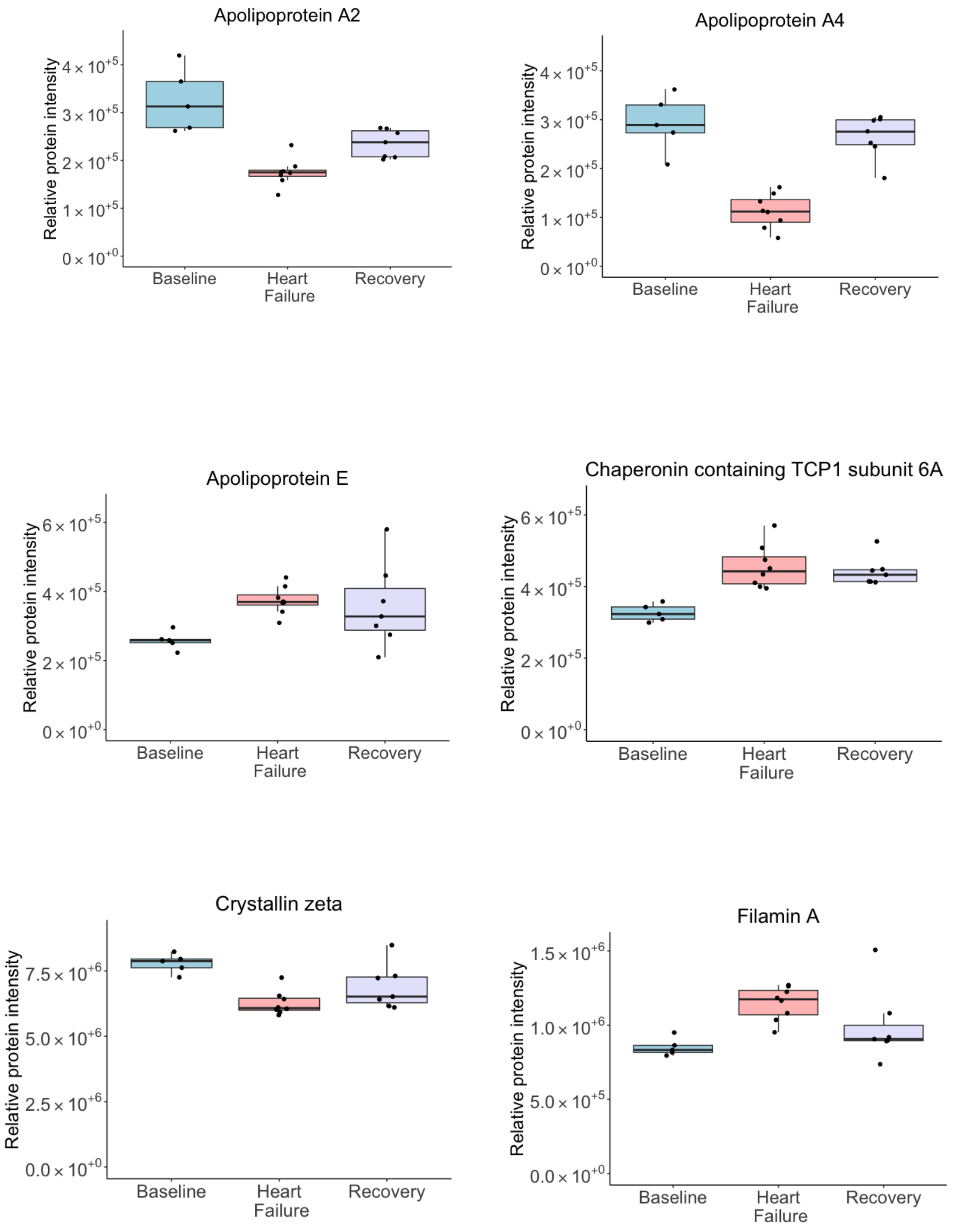

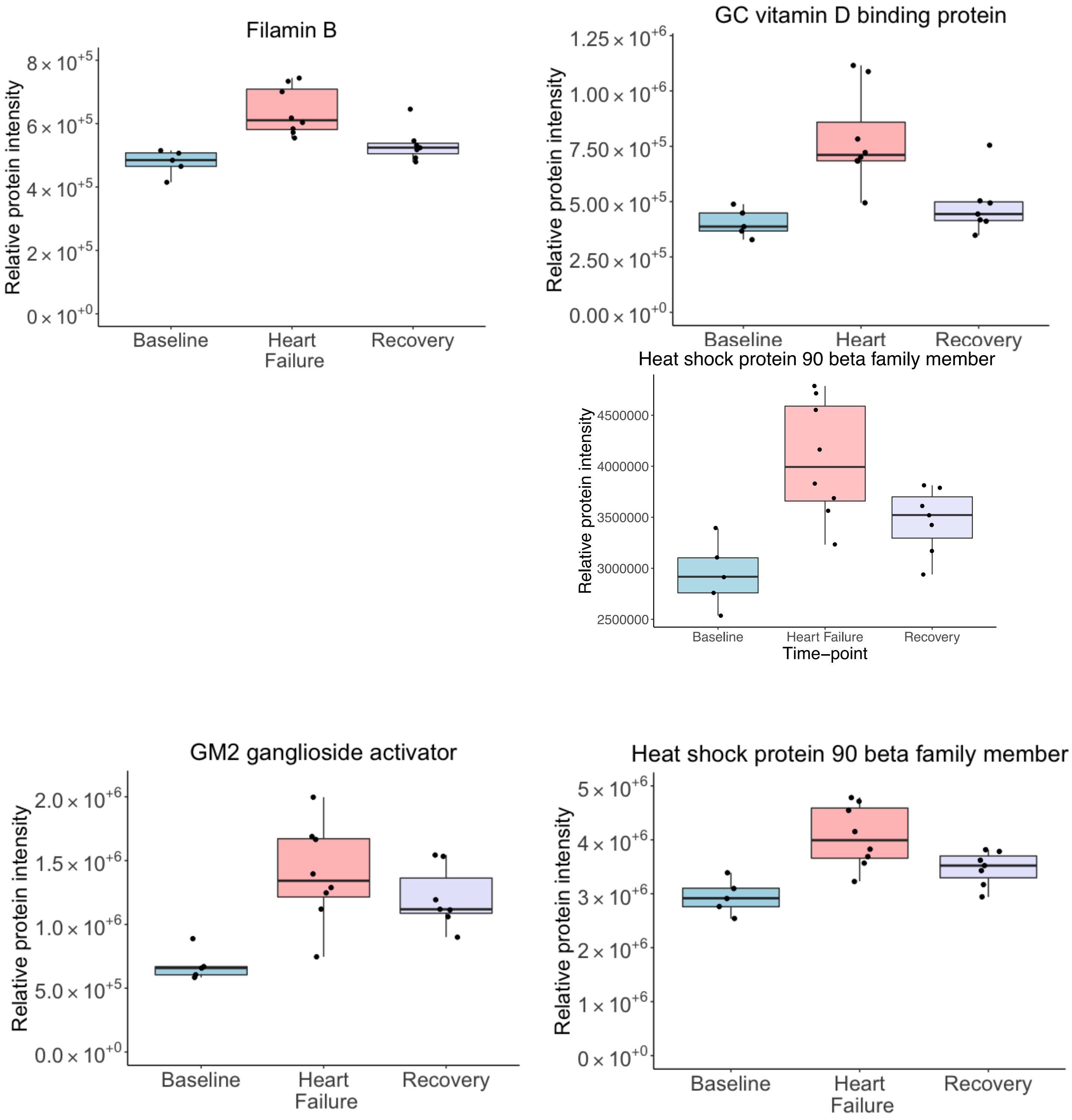

Filamin B
Relative protein intensity
8 × 10^+5
6 × 10^+5
4 × 10^+5
2 × 10^+5
0 × 10^+0
Baseline
Heart Failure
Recovery
GC vitamin D binding protein
Relative protein intensity
1.25 × 10^+6
1.00 × 10^+6
7.50 × 10^+5
5.00 × 10^+5
2.50 × 10^+5
0.00 × 10^+0
Baseline
Heart
Recovery
Heat shock protein 90 beta family member
Relative protein intensity
4500000
4000000
3500000
3000000
2500000
Baseline
Heart Failure
Recovery
Time−point
GM2 ganglioside activator
Relative protein intensity
2.0 × 10^+6
1.5 × 10^+6
1.0 × 10^+6
5.0 × 10^+5
0.0 × 10^+0
Baseline
Heart Failure
Recovery
Heat shock protein 90 beta family member
Relative protein intensity
5 × 10^+6
4 × 10^+6
3 × 10^+6
2 × 10^+6
1 × 10^+6
0 × 10^+0
Baseline
Heart Failure
Recovery

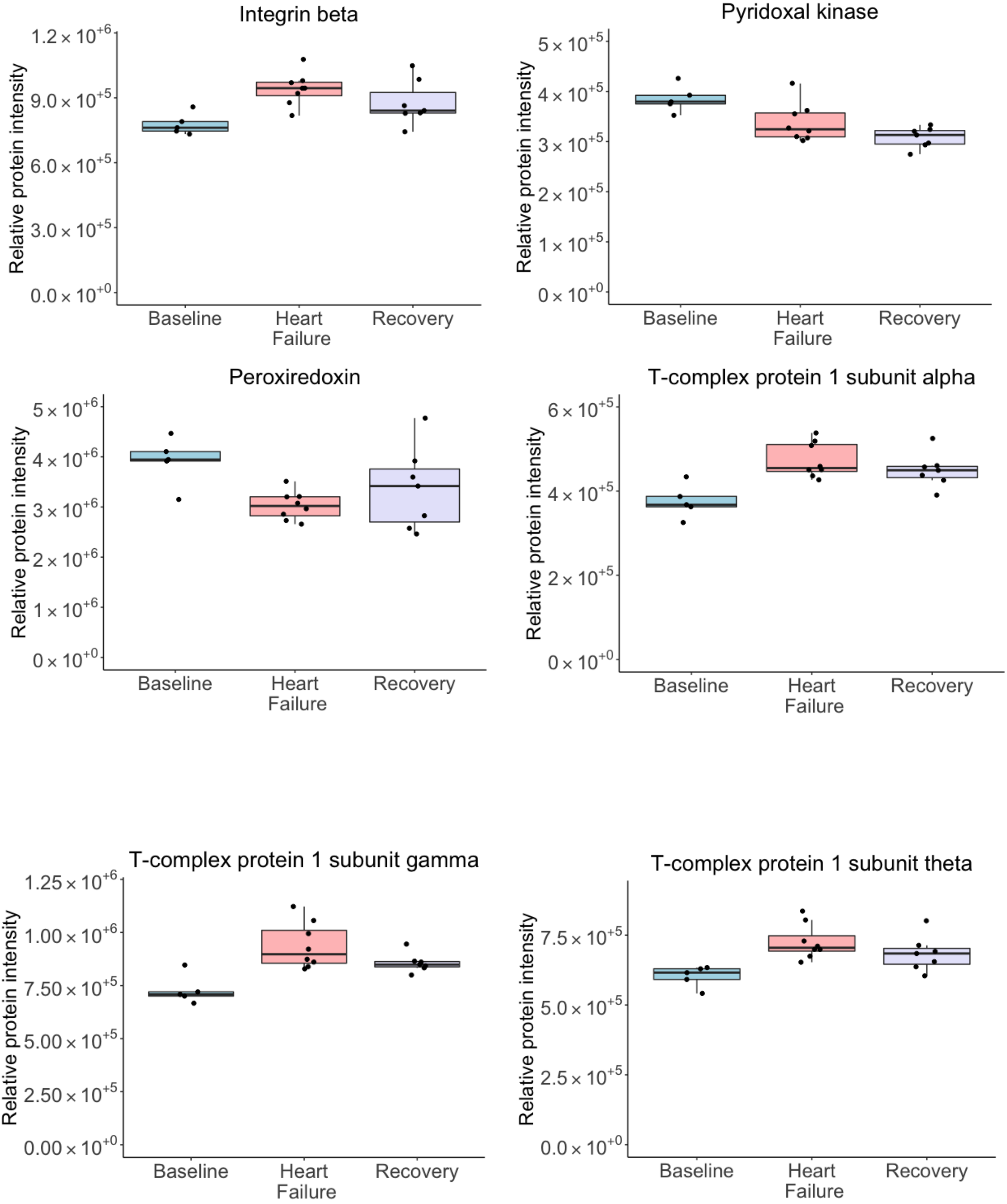
Integrin beta
Relative protein intensity
1.2 × 10+6
9.0 × 10+5
6.0 × 10+5
3.0 × 10+5
0.0 × 10+0
Baseline
Heart Failure
Recovery
Pyridoxal kinase
Relative protein intensity
5 × 10+5
4 × 10+5
3 × 10+5
2 × 10+5
1 × 10+5
0 × 10+0
Baseline
Heart Failure
Recovery
Peroxiredoxin
Relative protein intensity
5 × 10+6
4 × 10+6
3 × 10+6
2 × 10+6
1 × 10+6
0 × 10+0
Baseline
Heart Failure
Recovery
T-complex protein 1 subunit alpha
Relative protein intensity
6 × 10+5
4 × 10+5
2 × 10+5
0 × 10+0
Baseline
Heart Failure
Recovery
T-complex protein 1 subunit gamma
Relative protein intensity
1.25 × 10+6
1.00 × 10+6
7.50 × 10+5
5.00 × 10+5
2.50 × 10+5
0.00 × 10+0
Baseline
Heart Failure
Recovery
T-complex protein 1 subunit theta
Relative protein intensity
7.5 × 10+5
5.0 × 10+5
2.5 × 10+5
0.0 × 10+0
Baseline
Heart Failure
Recovery

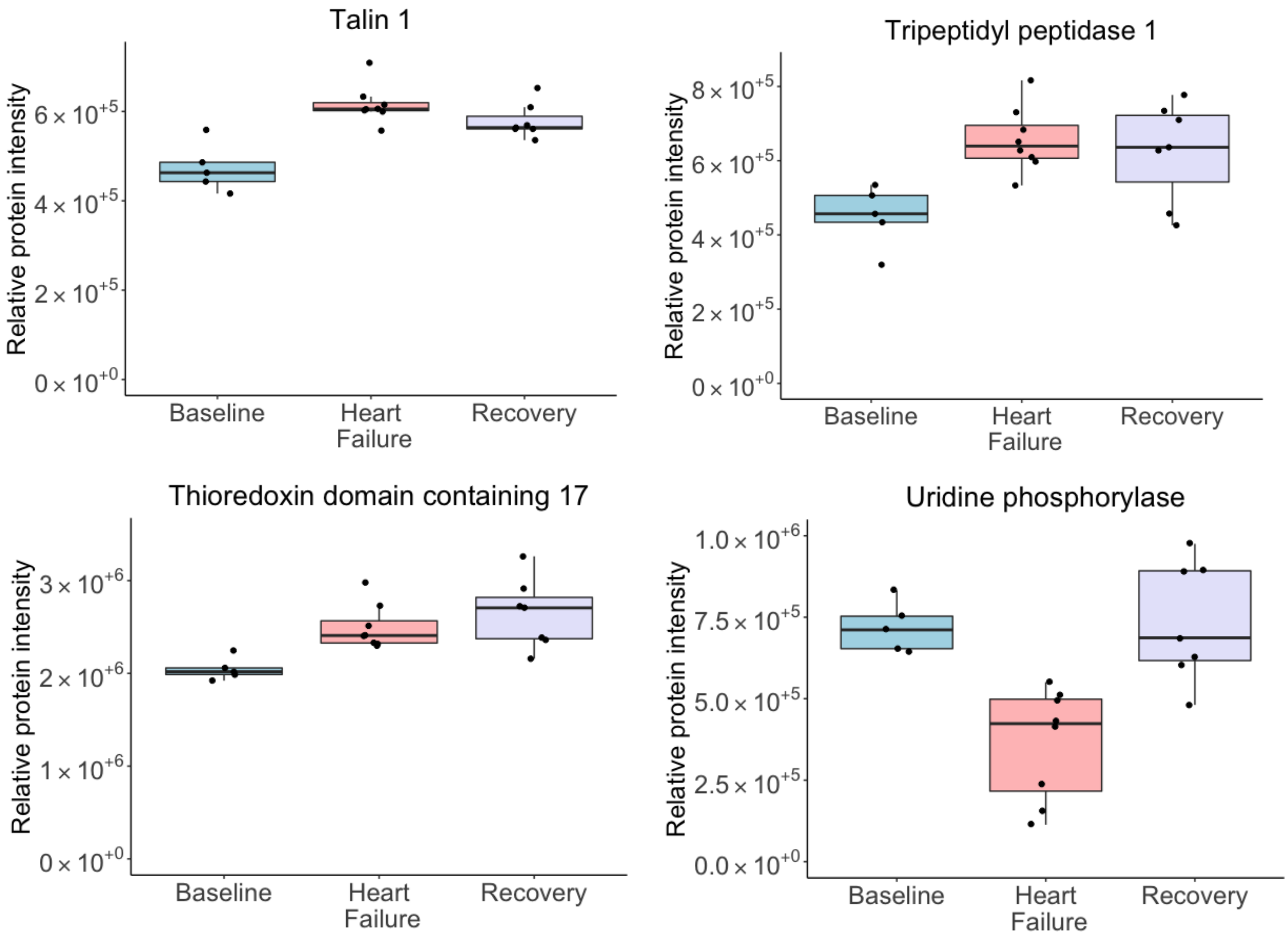

Figure S4. Correlation matrix of B-type natriuretic peptide (BNP), plasma renin activity, plasma creatinine, creatinine clearance and the 20 candidate protein biomarkers at (a) Baseline, (b) Heart Failure and (c) Recovery. Colour signifies the directionality of correlations, with red showing a negative correlation and blue showing a positive correlation. Colour intensities are proportional to the correlation coefficients (Spearman correlations, hierarchichal clustering method "Ward").

S4A Baseline

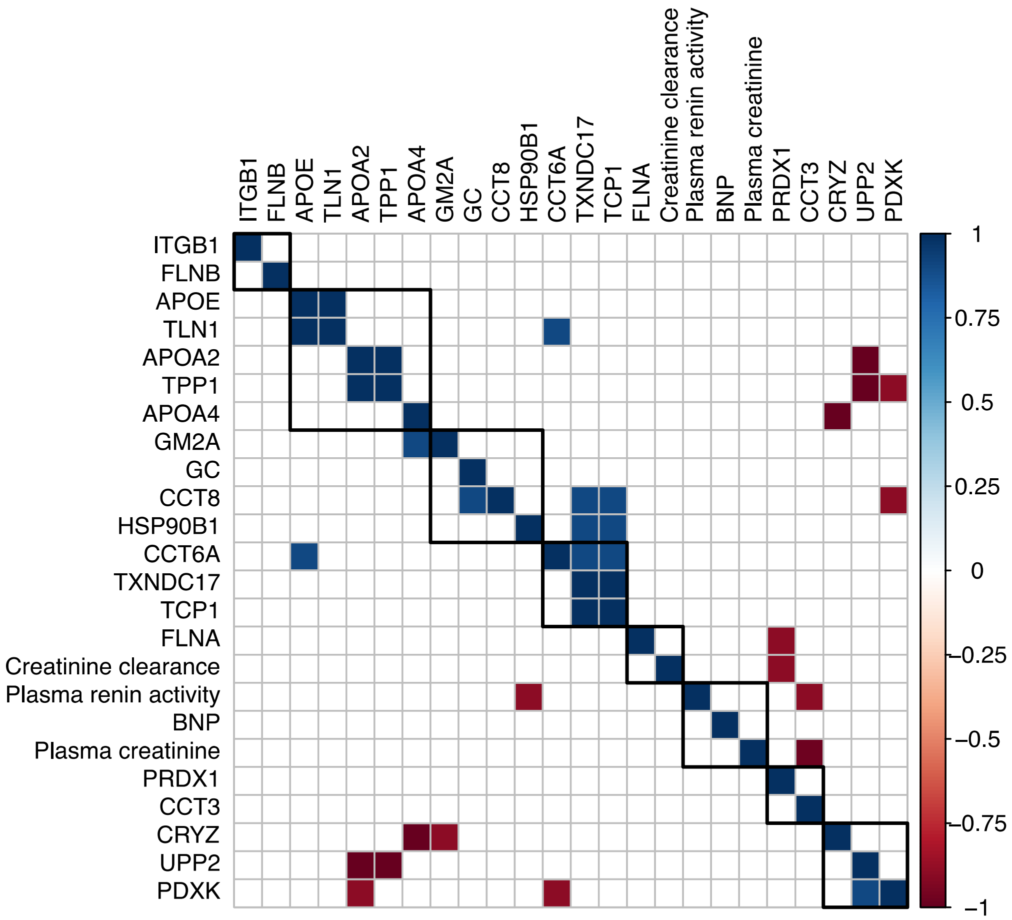

S4B Heart Failure

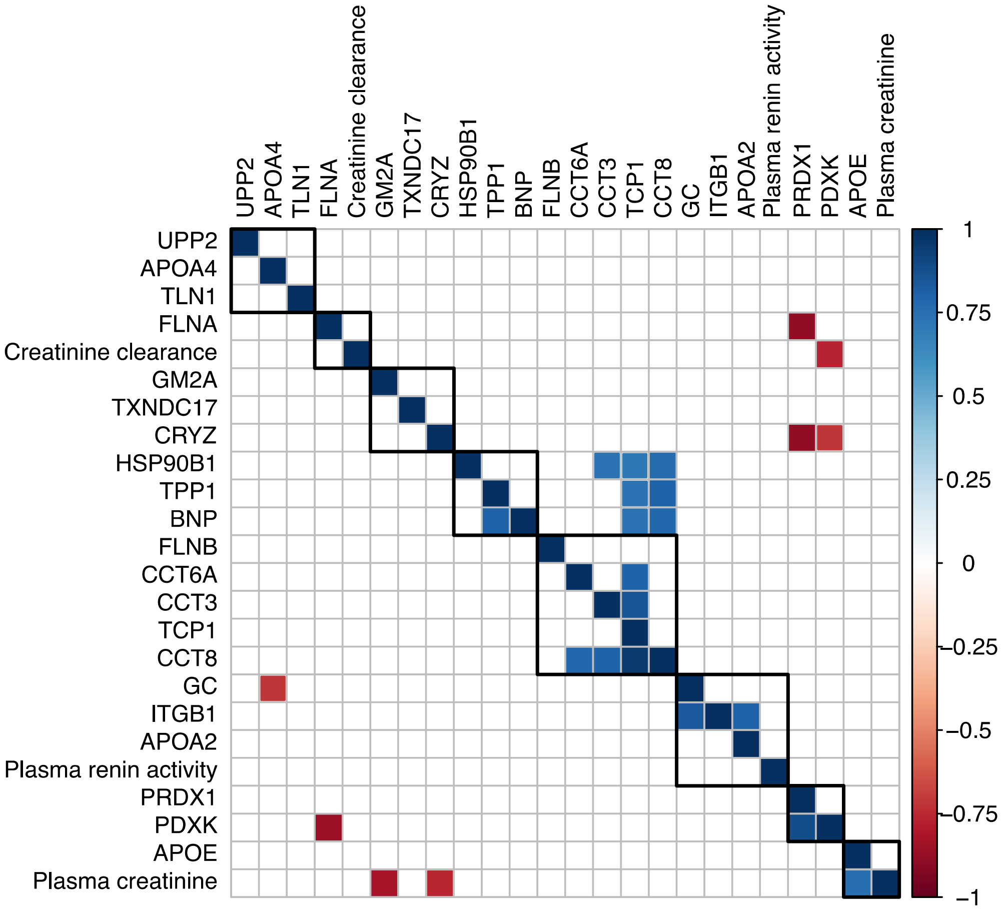

S4C Recovery

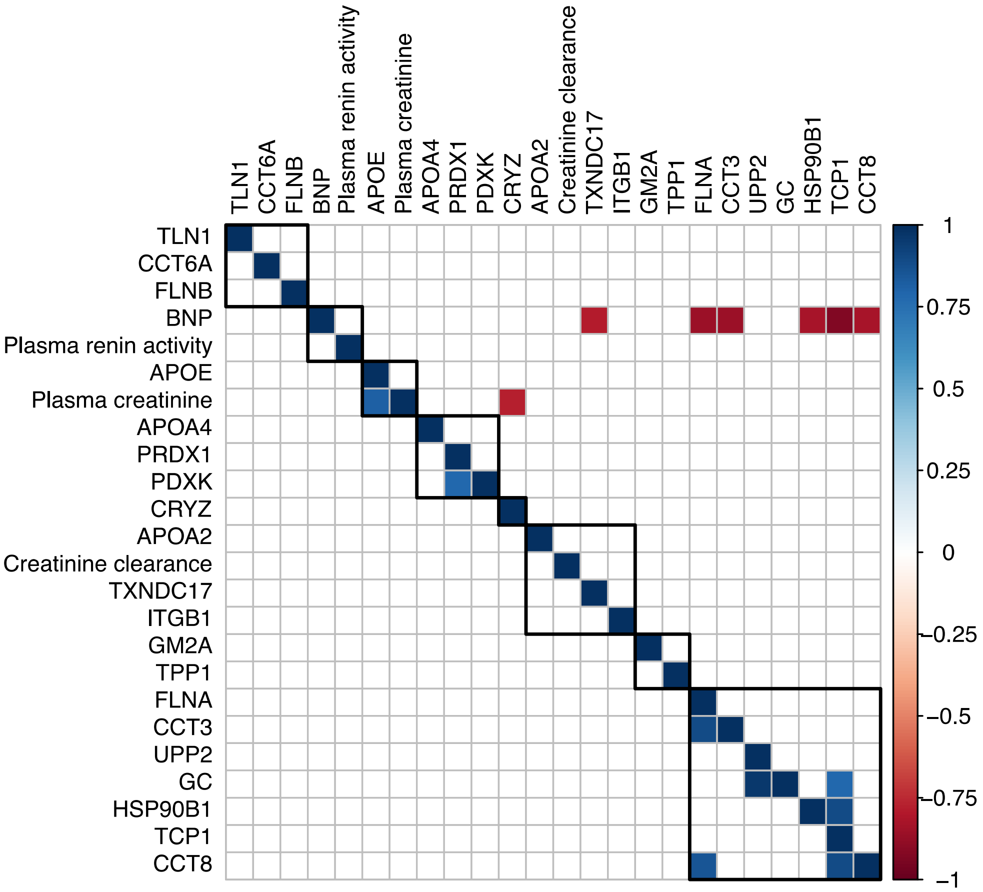

Table S2. Gene expression changes in kidney tissue with a fold-change ≥ 1.2 and adjusted p-value < 0.01 between any pair of timepoints (Baseline and ADHF, ADHF and Recovery form ADHF, Baseline and Recovery from ADHF). Protein abundance changes in kidney tissue with a fold-change of ≥ 1.2 and adjusted p-value < 0.05 between any pair of timepoints. Gene expression data obtained from Rademaker et. al. 2021 [20].

Protein	Gene	Time-point	Protein fold-change	Protein adjusted p-value	Gene fold-change	Gene adjusted p-value
Chaperonin containing TCP1 subunit 6A	*CCT6A*	B – HF	↑ 1.4	0.03	↑ 1.4	< 0.001
		B - R	↑ 1.35	0.02		
Filamin A	*FLNA*	B – HF	↑ 1.35	0.02		
		HF - R			↓ 1.73	<0.001
Filamin B	*FLNB*	B – HF	↑ 1.34	0.03		
		HF - R			↓ 1.34	0.004
Heat shock protein 90 beta family 1	*HSP90B1*	B – HF	↑ 1.38	0.04	↑ 1.37	0.006
		HF - R			↓ 1.42	0.003
Pyridoxal kinase	*PDXK*	HF-R	↓ 1.25	0.02	↓ 1.28	0.005

Figure S5. Scatter plots of protein fold-changes and fold-changes for the encoding genes between a) Baseline and Heart Failure, b) Heart Failure and Recovery and c) Baseline and Recovery. Proteins with an abundance fold-change of ≥1.2 (adjusted p-value < 0.05) for which the encoding gene also had a fold-change of ≥1.2 (adjusted p-value <0.01) between the same pair of timepoints are plotted in red and labeled. Gene expression data obtained from Rademaker et. al. 2021 [20].

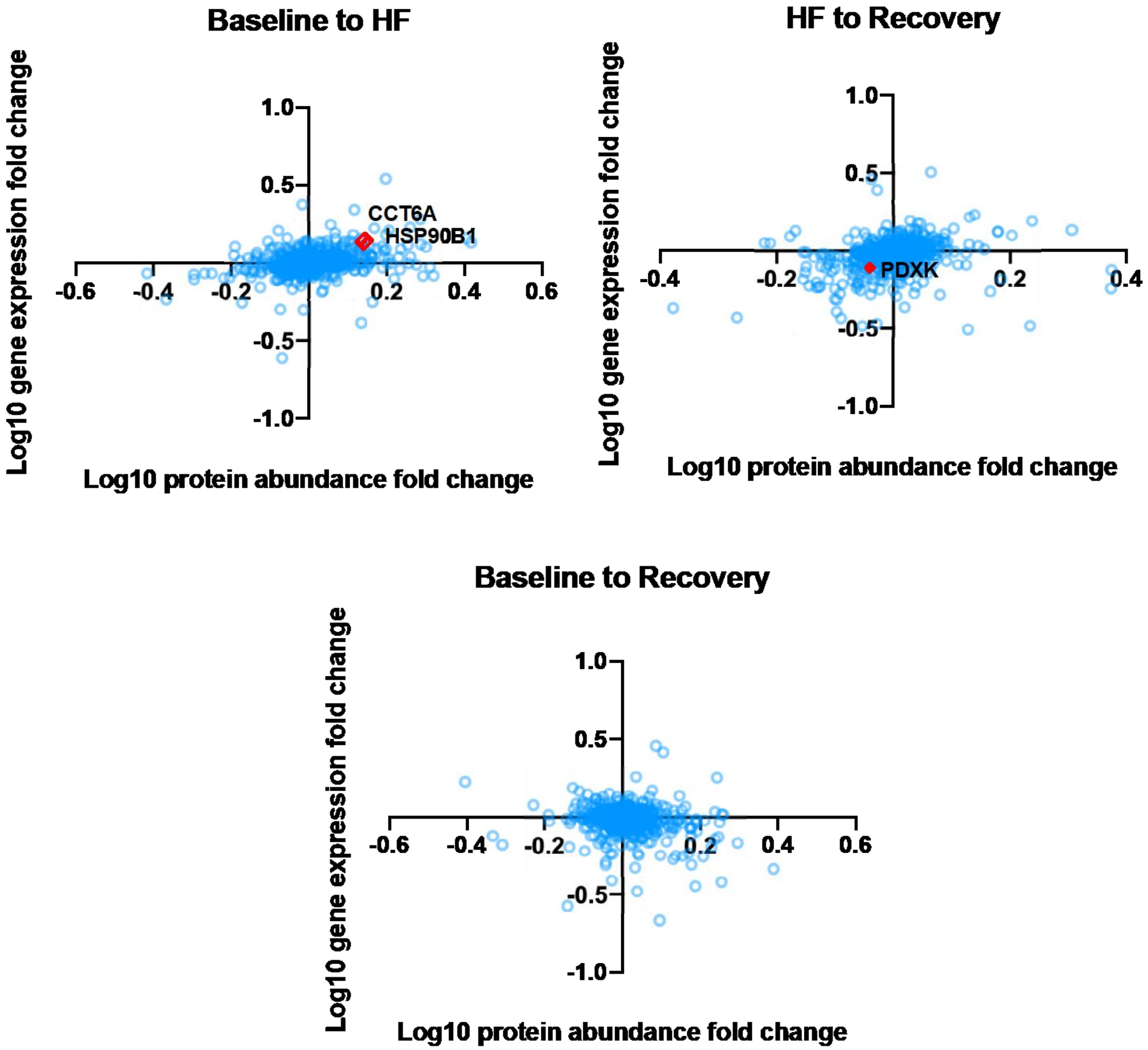

Figure S6. Study timelines for the preparation of (a) control sheep ('Baseline' group, n=5), (b) paced sheep ('Heart Failure' group, n=8) and (c) sheep recovered from heart failure ('Recovery' group, n=7). Created using biorender.com.

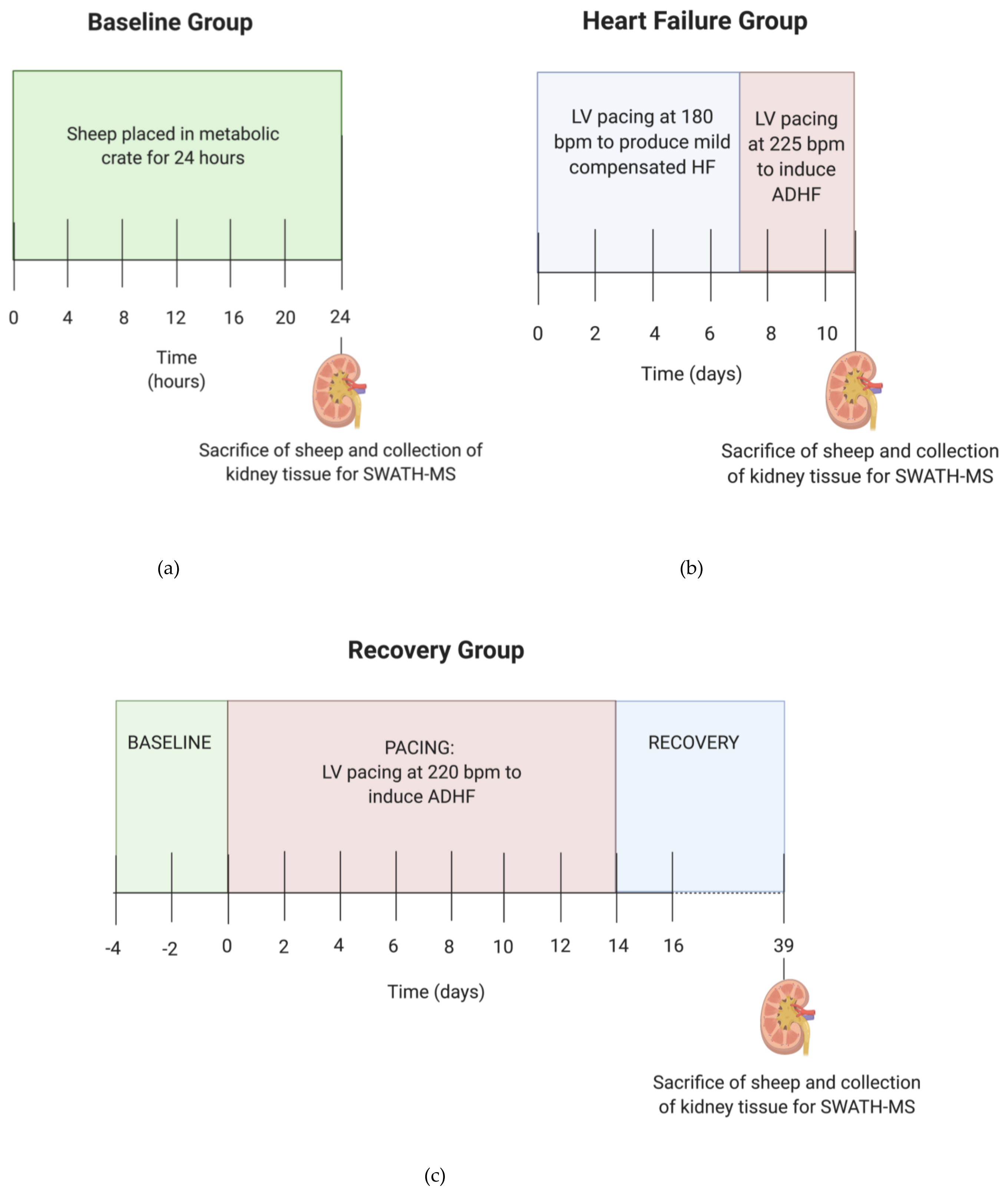

20. Rademaker, M.T.,Pilbrow, A.P.,Ellmers, L.J.,Palmer, S.C.,Davidson, T.,Mbikou, P., et al. Acute Decompensated Heart Failure and the Kidney: Physiological, Histological and Transcriptomic Responses to Development and Recovery. *J Am Heart Assoc*. **2021**;*10*(18):e021312.
