# Supplementary material for: Identifying Candidate Protein Markers of Acute Kidney Injury in Acute Decompensated Heart Failure
Source: Int J Mol Sci. 2022 Jan 17;23(2):1009. doi: 10.3390/ijms23021009 (PMC8778509; doi:10.3390/ijms23021009)
Supplement: Supplementary file 1 [file ijms-23-01009-s001.zip › Supplementary Methods Templeton et al.pdf]

Supplementary methods

Study protocol for paced sheep [52, 53]

Hemodynamic measurements (including cardiac output, mean arterial pressure, central venous pressure and left atrial pressure) were measured using an on-line data acquisition system (PowerLab Systems; ADInstruments, New Zealand) prior to the initiation of pacing (baseline), during pacing, and following the termination of pacing in the "Recovery" group sheep until the protocol was complete. Blood samples for assay of plasma hormones (ANP, BNP, PRA) and biochemical measurements (creatinine) were drawn into containers on ice, centrifuged at 4°C within 5 min, then stored at -80°C until analyzed. Urine was collected daily for measurement of volume and creatinine excretion. Following cessation of pacing in "Recovery" sheep, these measurements continued daily for one week, and then every 2-3 days until the protocol was complete. Creatinine clearance (mL/min) was calculated as urine creatinine concentration x volume/plasma creatinine (measured 2-hourly and daily).

Sample preparation

Peptides were loaded onto C18 resin (SNS SS18V-L, The Nest Group Inc., Ipswich) and salts and buffers washed three times using 200 μL 0.2% TFA with a stepped-centrifugation protocol (50 g, 110 g, 200 g and 400 g for 1 minute each, followed by 750 g for 2 minutes). Peptides were eluted using a high-organic mobile phase (0.2% formic acid in 75% ACN) using the same centrifugation protocol (50 g, 110 g, 200 g and 400 g for 1 minute each, followed by 750 g for 2 minutes).

Mass Spectrometry

Gradient: The following linear gradient steps of solvent B were used across all samples at a flow rate of 300 nL/min.: loading at 5% B, 3 min at 5% B, 90 min increase to 25% B, 10 min increase to 40% B, 10 min increase to 95% B, 1 min at 95% B, 1 min decrease to 5% B, 5 min at 5% B.

Data dependent acquisition protocol: For each sample, an MS1 survey scan was run (400 ms) followed by 22 MS2 scans in which the most abundant peptides were fragmented, covering a precursor mass range of 400-1300 mass to charge (m/z). All high-sensitivity fragment ion scans used an accumulation time of 140 ms, resulting in a total cycle time of ~3.5 seconds for all 23 scans.

SWATH-MS protocol: First, an MS survey scan was acquired at 100 ms accumulation time, followed by a total of 34 SWATH-MS fragment ion scans with overlapping m/z windows (1 m/z for the window overlap), covering a precursor mass range of 400-1250 m/z. SWATH-MS spectra were collected from 100-1600 m/z. Collision energy spread (CE) was automatically optimized for each window using a CE spread of 15 eV. All high-sensitivity fragment ion scans used an accumulation time of 96 ms, resulting in a total cycle time of ~3.4 seconds for all 35 scans.

Spectral library generation

ProteinPilot (v4.5, AB Sciex) was used to process DDA data for library construction. AB Sciex *wiff DDA files were searched against the a concatenated target/decoy [66] version of the Ovis aries (27,638 entries, 99.8%), Ovis aries musimon (46 entries, 0.2%) and Ovis aries platyura (1 entry, <0.01%) sheep proteomes from UniProtKB [67] TrEMBL (27,685 target sequences, accessed 22 July 2020). Searches were performed using the Paragon™ algorithm (v4.5.0.0) using the following search

parameters: i) sample type: identification; ii) cys-alkylation: iodoacetamide; iii) digestion: trypsin; iv) instrument: TripleTOF 5600; v) special factors: none; vi) ID focus: biological modifications; amino acid substitutions; vii) search effort: thorough ID; and viii) detected protein threshold: > 0.05 (10.0%).

Retention time alignment

Table A1. Peptides used for retention time alignment in SWATH data processing

Protein	Peptide	Expected RT
Biognosys iRT protein	ADVTPADFSEWSK	62.53
	TPVITGAPYEYR	45.48
	GAGSSEPVTGLDAK	21.74
	YILAGVENSK	33.14
	LGGNEQVTR	9.22
	TPVISGGPYEYR	42.31
LDL receptor related protein OS=Ovis aries, OX=9940, GN=LRPAP1, PE=4, SV=1	LLTVNPWLTQVR	77.03
Tubulin alpha chain, OS=Ovis aries, OX=9940, GN=TUBA4A, PE=2, SV=1	AVFVDLEPTVIDEIR	92.83

Construction of AKI gene list

To construct the gene list from Harmonizome [26], genes associated with AKI from the following databases were selected: GAD Gene-Disease Associations (n=2), HuGE Navigator Gene-Phenotype Associations (n=21), and CTD Gene-Disease Associations with a standardized value > 2 (equivalent to p < 0.01) were selected (n=806; 15 genes appearing in more than one dataset). This gene list (n=814) was combined with a list of genes associated with AKI from the Ingenuity Pathway Analysis knowledgebase (n=94) to obtain the final list of 884 unique genes.

References

26. Rouillard, A.D., Gundersen, G.W., Fernandez, N.F., Wang, Z., Monteiro, C.D., McDermott, M.G., et al. The harmonizome: a collection of processed datasets gathered to serve and mine knowledge about genes and proteins. *Database (Oxford)*. **2016**;*2016*.

52. Fitzpatrick, M.A.,Nicholls, M.G.,Espiner, E.A.,Ikram, H.,Bagshaw, P., Yandle, T.G. Neurohormonal changes during onset and offset of ovine heart failure: role of ANP. *American Journal of Physiology*. **1989**;*256*:H1052-9.

53. Rademaker, M.T.,Charles, C.J.,Espiner, E.A.,Frampton, C.M.,Nicholls, M.G., Richards, A.M. Natriuretic peptide responses to acute and chronic ventricular pacing in sheep. *Am J Physiol*. **1996**;*270*(2 Pt 2):H594-602.

66. Elias, J.E., Gygi, S.P. Target-decoy search strategy for mass spectrometry-based proteomics. *Methods Mol Biol*. **2010**;604:55-71

67. Apweiler, R., et al. UniProt: the Universal Protein knowledgebase. *Nucleic Acids Res.* **2004**;32(Database issue):D115-119
